# Fatigue Crack Propagation Properties of Ordinary Plain Concrete Under Three-Point Loading

**DOI:** 10.3390/ma18245554

**Published:** 2025-12-11

**Authors:** Huating Chen, Jiapeng Song, Dewang Li

**Affiliations:** 1 State Key Laboratory of Bridge Safety and Resilience, Beijing University of Technology, Beijing 100124, China; 2 Department of Civil Engineering, Beijing University of Technology, Beijing 100124, China; jiapengsong@emails.bjut.edu.cn; 3 Guangzhou Expressway Co., Ltd., Guangzhou 510335, China; lidewang.gjtjt@gmail.com

**Keywords:** crack growth rate test, single-edge notched beam, ordinary concrete, fatigue crack propagation properties, Paris Law, Forman Equation

## Abstract

To obtain fatigue crack propagation properties of ordinary concrete commonly employed in bridge construction, 48 replicate single-edge notched beam specimens were fabricated using C50 plain concrete. Twelve of these were subjected to monotonic loading to determine their static capacity; the remaining 36 were fatigue-loaded with various combinations of maximum stress level and stress ratio under three-point bending. Visual observation, strain gauges, and the compliance method were used to determine the evolution of crack length during fatigue loading. The fatigue crack growth rates were then evaluated for each specimen using linear regression. This study shows that the fracture surface under fatigue loading exhibits greater zigzagging than under monotonic loading, with multiple microcracks coalescing. The elastic compliance method captures the three-stage development of fatigue crack well, and the derived equivalent crack size is consistently smaller than surface measurements. Significant scatter exists in the test data; however, the crack growth rate and stress intensity factor range follow a straight line on logarithmic scales, indicating that the Paris Law applies to plain concrete. The slope and intercept of C50 concrete, based on 27 fatigue-failed specimens, follow a Normal distribution, with means of 16.46 and −24.81 (in N-mm units), and coefficients of variation of 0.38 and −0.38, respectively. The corresponding mean and coefficient of variation for slope and intercept by the Forman Equation are 14.80 and 0.42 and −21.18 and −0.44, respectively. The fatigue crack in C50 concrete of this study shows a faster growth rate (46.7% larger slope) than that in lower-strength concrete in the literature. With further research needs identified, this study contributes to a better understanding of the fatigue crack growth properties of ordinary structural concrete, providing valuable information for fatigue assessment and service-life extension of existing concrete bridges.

## 1. Introduction

Fatigue performance of concrete bridge girders, bridge decks, road pavements, and wind turbine foundations has gained increasing attention in recent years [1]. One inherent disadvantage of structural concrete is the presence of initial imperfections, such as microcracks, which can be caused by internal and external factors, including hydration heat, thermal stress, and restrained shrinkage [2]. The continuous development of these defects into macrocracks under the action of alternating vehicle, temperature, water pressure, and wind loads, called fatigue, could eventually lead to the fracture of structural components [3,4]. Fatigue crack propagation properties are therefore the basis for fatigue life evaluation and the prerequisite for improving the safety and durability of existing concrete bridges and structures [4].

The fatigue crack propagation behavior of concrete is typically characterized through fatigue crack growth rate experiments conducted on small-scale notched specimens. Several specimen configurations, such as bending beams and splitting wedges, have been employed for this purpose [5,6]. Owing to its material efficiency and testing simplicity, the single-edge notched beam (SENB) specimen subjected to three-point bending has become the most widely adopted configuration, particularly in flexural studies [4]. Since the 1980s, extensive experimental investigations have been performed to examine the fatigue crack propagation characteristics of concrete [7,8]. It is generally agreed that fatigue crack growth in concrete can be divided into three distinctive stages: rapid macrocrack formation, stable crack extension, and unstable crack propagation. Stable crack extension can be observed in 60~80% of fatigue loading cycles, and the crack propagation rate in the last stage is very high, eventually leading to fracture [8]. The effects of many relevant parameters, such as specimen size [9,10,11,12], notch-to-height ratio [13], coarse aggregate size [14], loading frequency [15], and variable amplitude fatigue loading [16,17,18] on the fatigue crack propagation behavior of concrete have been extensively investigated in the past decades. Recent studies indicate that the primary focus of research has progressively shifted from conventional concrete towards high-performance concrete [10,19,20,21], self-compacting concrete [22], pervious concrete [23], and the effects of fiber reinforcement [19,20,21,24,25], rubber content [26], and alkali activation [27]. In short, earlier fatigue studies mainly investigated lower-strength ordinary concrete (<C40) available at the time. As modern concrete technology has changed significantly, C40~C60 has become common. Since current research focuses more on innovative high-strength (>C60) composite concrete, fatigue crack growth properties of C40~C60 ordinary concrete widely used in bridge engineering remain insufficiently studied.

Detecting and monitoring fatigue crack size at various stages of cyclic fatigue loading is crucial to determining the fatigue crack growth rate. In addition to naked-eye observation, various methods such as the compliance method, resistance strain gauges [12], photoelastic coating method [28], acoustic emission (AE) [14,22,23,26,29], and digital image correlation (DIC) [14,20,24,30,31] have been explored to monitor fatigue crack size. Nevertheless, the compliance method remains the most widely employed due to its ease of application for crack growth monitoring up to a relative crack size of 0.60 [7]. The compliance method uses a compliance calibration curve to estimate crack size from specimen compliance, which is a synonym for flexibility—the reciprocal of stiffness—and is conceptually defined as the crack mouth opening displacement (CMOD) divided by the load *P*. However, the method to calculate compliance under fatigue loading is confusing. For a specific fatigue loading cycle, it can be calculated as the ratio of CMOD to *P* at the maximum fatigue load, as the secant value (CMOD/*P*), or as the tangent value on the loading or unloading branch [5,8]. For a given compliance calibration curve and the various methods used to obtain it, different definitions of compliance yield different crack sizes and fitted crack propagation material constants. Therefore, it is necessary to define the compliance calibration curve clearly and to investigate the applicability and differences among various calculation methods for obtaining real-time compliance during fatigue loading.

Structural concrete, as a quasi-brittle material, is different from structural steel in the sense of heterogeneous material composition and the existence of a fracture process zone in front of the crack tip [32,33]. Given the nonlinear effect of the fracture process zone on the effective crack size, empirical equations based on the stress intensity factor *K* of an equivalent crack are a practical method for describing fatigue crack propagation in concrete [5,8]. Among them, the Paris’ law, which relates the steady fatigue crack growth rate d*a*/d*N* to the stress intensity factor range Δ*K*, represents the most classical and widely recognized model [34]. Forman et al. proposed a modified form of Paris Law that accounts for the stress ratio *R* and instability when the maximum stress intensity *K*_max_ approaches the material’s fracture toughness *K*_IC_ [35]. Afterward, various scholars have modified Paris Law to explain the discreteness of concrete fatigue tests [9,10,11,12]. However, previous studies on whether Paris Law and Forman Equation are valid for describing the fatigue crack propagation properties of plain concrete are not always consistent [4,8,12,13]. More research is required to assess the applicability of these equations in engineering practice.

An extensive experimental program was conducted in the Engineering Mechanics Laboratory at Beijing University of Technology to characterize the fatigue and fracture properties of ordinary plain C50 concrete, which is widely used in bridge engineering across China. In addition to the fatigue life S-N curves of intact specimens and their statistical characteristics reported in Reference [36], and the fracture toughness of SENB specimens under static loading presented in Reference [37], this paper specifically investigates the fatigue crack growth rate behavior under cyclic loading. The relevant results from the static fracture test were briefly described, and readers could refer to our previous publication [37] for more details. Fatigue crack propagation properties of plain concrete were obtained from three-point bending tests of 36 SENB specimens. Longitudinal concrete strain was recorded during the fatigue crack growth rate test to obtain a complete load–CMOD curve for deriving fatigue crack size using the compliance method. In addition, crack size was measured through visual observation and strain gauges affixed to the specimen’s side surface. Fatigue crack propagation parameters were derived from fatigue crack growth rate test data using the classical Paris Law and Forman Equation, and the outcomes of both approaches were comparatively analyzed. The fatigue crack propagation results were further compared with data reported in the literature for lower-strength ordinary concrete. A distinctive feature of this study lies in its comprehensive evaluation of three fatigue crack size measurement methods and two fatigue crack growth rate models to deduce fatigue crack propagation properties. The practical implications of this study concern the assessment of bridge performance and the service life extension of existing concrete structures.

## 2. Materials and Methods

### 2.1. Concrete and Mix Design

A ready-mixed C50-grade ordinary concrete was selected for the experimental program because of its extensive use in bridge construction. Table 1 presents the concrete mixture, with specific components and proportions formulated in accordance with national standard specifications. There were four main ingredients—cement, sand, stone, and water—and the mix ratio was designed as 1:1.93:3.02:0.46 to achieve the desired strength and workability. The aggregate-to-cement ratio was 4.95, and approximately 60% of the total aggregate was coarse aggregate. The use of 42.5 Portland cement, medium natural sand with a fineness modulus of 2.4, along with rubble and cobble gravel sized between 5 and 25 mm, was determined by their availability and suitability for bridge engineering. The desired slump of the fresh concrete mixture was aimed at 180 ± 20 mm, and a 1.99% high-performance water-reducing agent, known as STD-PCS (a superplasticizer made from polycarboxylic acid, produced by Tianjin Steady Industrial Development Co., Ltd., Tianjin, China), was incorporated to enhance workability and minimize shrinkage. In addition, 115 kg of admixtures (including mineral powder and fly ash) were included per cubic meter of concrete to improve compressive strength and impermeability, and to reduce hydration heat and cement consumption.

The same batch of concrete was used to produce fatigue crack growth rate test specimens in this paper and those fracture toughness test specimens in Reference [37].

### 2.2. Specimen Fabrication

**Figure 1:**
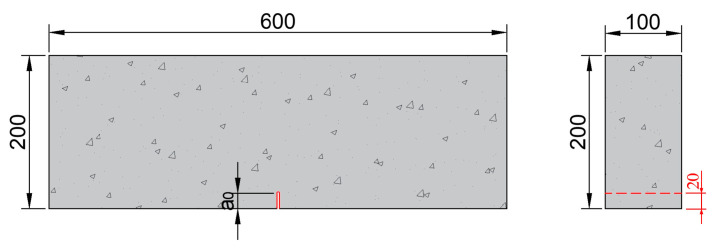
Schematic representation showing the single-edge notched beam (SENB) specimens with a notch depth *a*_0_ of 20 mm (all dimensions in mm). The prefabricated notch is enlarged for illustrative purposes.

Single-edge notched beam (SENB) specimens were used in the fatigue crack growth rate test. The specimen dimensions are 100 mm × 200 mm × 600 mm, as shown in Figure 1. The test was conducted to assess the concrete’s resistance to stable crack propagation, which is a key factor to consider in bridge engineering. The specimen height of 200 mm corresponds to the typical lower limit used in bridge deck applications. Through-thickness straight notches of shallow depth were introduced at the midspan section of the bottom surface of SENB specimens. A total of 36 specimens were fabricated for testing.

**Figure 2:**
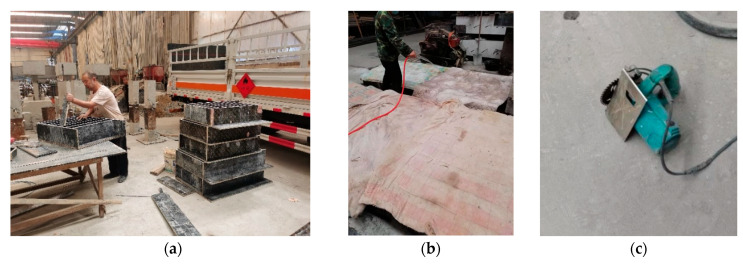
Fabrication of single-edge notched beam (SENB) specimens: (**a**) Wooden formwork; (**b**) Spraying water curing; (**c**) Cutting tool for preparing shallow notches [37].

All specimens were cast using assembled wooden formwork to reduce the effects of concrete shrinkage and creep, as shown in Figure 2a. After casting, they were covered with a polyethylene sheet and cured for 28 days under standard conditions (22 °C and 95% relative humidity). Subsequently, the specimens were stored indoors without special treatment at a room temperature of 17~27 °C and a relative humidity of 30~80%. At the time of testing, the specimens had an age ranging from 60 to 90 days. Figure 2b shows a photograph of the specimens during the curing process.

All specimens had an initial notch size of 20 mm, with a notch depth-to-specimen height ratio α_0_ of 0.1. The prefabricated two mm wide notches in the SENB specimens were produced using a concrete cutting machine, as shown in Figure 2c, to achieve the specified notch depth after casting and formwork removal. The cutting process was stopped 2~3 mm before reaching the target notch depth to minimize potential damage from the saw teeth. A positioning plate was used to ensure consistent notch depth across the specimen thickness.

To verify the concrete grade, nine 150 mm × 150 mm × 150 mm companion concrete cubes, cast from the same batch and cured under identical conditions to the SENB specimens, were tested for compressive strength after 30 days of curing.

### 2.3. Testing Setups

**Figure 3:**
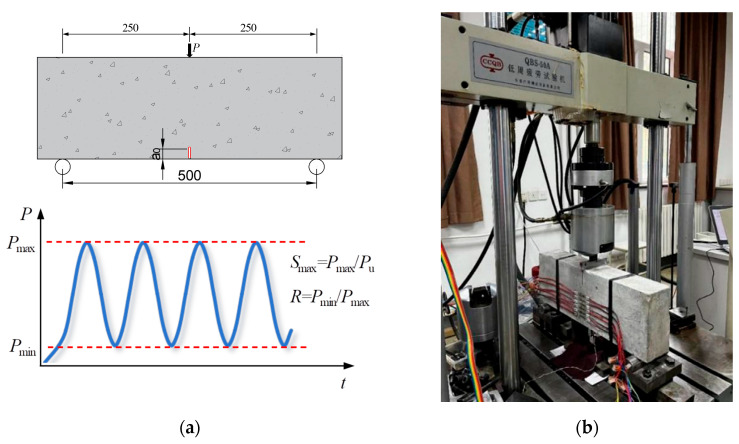
Three-point bending fatigue crack growth rate test: (**a**) Schematic illustration of the test setup (dimensions in mm); (**b**) Photograph of the experimental setup with the fatigue testing machine.

All plain concrete SENB specimens were tested under three-point bending conditions with a span-to-height ratio *S*/*H* of 2.5, as shown in the schematic illustration in Figure 3a. Altogether, 36 specimens were loaded cyclically by a low-cycle fatigue testing machine, as shown in Figure 3b.

The fatigue test was performed using a QBS electro-hydraulic servo-controlled universal testing system. The testing device made by Changchun Qianbang Testing Equipment Co., Ltd. (Changchun, China) could handle a maximum load of 50 kN and load at a frequency between 0.1 and 10 Hz, as demonstrated in Figure 3b.

A fatigue loading matrix with various combinations of maximum stress level and stress ratio was established. The stress level was defined as the ratio of the applied fatigue load to the static ultimate load *P*_u_. The ultimate load-carrying capacity *P*_u_, which was used to determine the fatigue loads, was obtained from a previous statically loaded fracture test [37]. The stress ratio *R* is the ratio between the minimum fatigue load *P*_min_ and the maximum fatigue load *P*_max_. *S*_max_ of 0.80, 0.85, and 0.90, and *R* of 0.1 and 0.5 (representing service conditions with relatively small and large mean stress, respectively) were considered in the test program, based on the standard test range reported in the literature survey and previous experience with intact beam specimens [36]. A sinusoidal load with constant amplitude was applied to maintain the desired stress levels. All specimens were tested at frequencies of up to 5 Hz. The specimens were aligned correctly on the fatigue testing system, and the loading parameters were calibrated to achieve the required fatigue load waveform and frequency. Details of the fatigue loading matrix for the fatigue crack growth rate test are presented in Table 2. The specimen is designated S-a-b-i, where S denotes stress control, a denotes the design maximum stress level, b denotes the design stress ratio, and i denotes the serial number within a fatigue test series.

The runout limit is set as the maximum number of cycles that can be applied within one day without machine stoppage and restart, approximately 100,000 fatigue loading cycles, to prevent any possible changes in concrete fatigue properties due to cycle intervals or disruptions.

### 2.4. Measurement and Instrumentation

**Figure 4:**
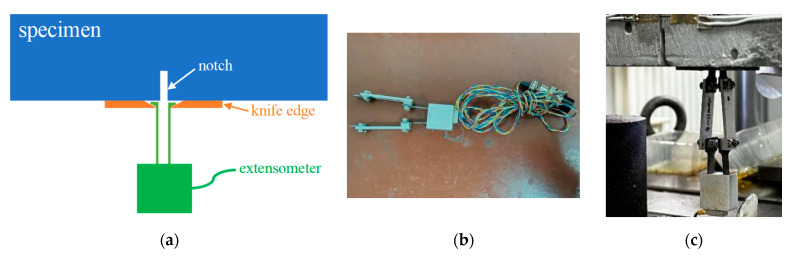
Arrangement of extensometer gauge [37]: (**a**) Scheme of CMOD measurement; (**b**) Photo of the extensometer; (**c**) Photo of mounted extensometer.

The testing machine automatically recorded actuator load and displacement data, displaying a real-time load–displacement curve for test monitoring. To continuously measure the crack mouth opening displacement (CMOD) during the fatigue crack growth rate test, an extensometer was installed on the specimen’s bottom surface across the notch opening using a pair of knife edges. The YYJ-(–2)-5/6 extensometer, manufactured by NCS Testing Technology Co., Ltd. (Beijing, China), featured a nominal gauge length of 6 mm, a measurement range of −2 to 5 mm, and a resolution of 0.001 mm. Figure 4 illustrates the arrangement of the extensometer.

For crack size measurement, three methods were used in this study: naked-eye observation with a microscope, resistance strain gauges, and the compliance method. Their accuracy, precision, and usefulness relative to one another can be validated on specimens simultaneously measured by more than one method.

**Figure 5:**
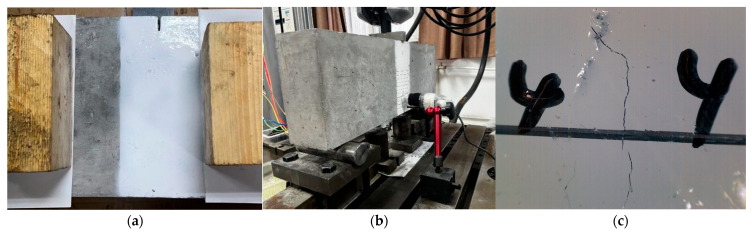
Visual observation of fatigue crack by an electronic microscope (maximum magnification of 240×): (**a**) White lacquer on the specimen’s side surfaces; (**b**) Electronic microscope and its setup [37]; (**c**) Observed fatigue crack by the microscope.

In terms of visual observation, a macroscopic crack that could be easily observed with the naked eye tends to break suddenly for ordinary plain concrete. In contrast, small fatigue cracks in the stable propagation stage are difficult to observe with the naked eye. An electronic portable microscope, the MDA2000 (manufactured by Hangzhou Future Optics Sci & Tech Co., Ltd., Hangzhou, China), with a maximum magnification of 240×, was employed to observe microscopic crack development, as shown in Figure 5b. The USB digital microscope was equipped with a 2.0 MP sensor and provided a maximum image resolution of 1600 × 1200 pixels. A coating of white, matte lacquer was applied to the side surfaces of the specimen in the anticipated crack propagation region to enhance crack visibility. Horizontal locating lines at 10 mm apart were drawn on the white surface after the paint had dried thoroughly. The microscope was then supported on an in-house camera stand, initially focusing on the prefabricated notch tip. Live images and enlarged views were shown on a computer screen, and the camera stand was adjusted periodically to maintain focus on the fatigue crack tip. Visual observation of a fatigue crack, aided by a microscope, is shown in Figure 5.

**Figure 6:**
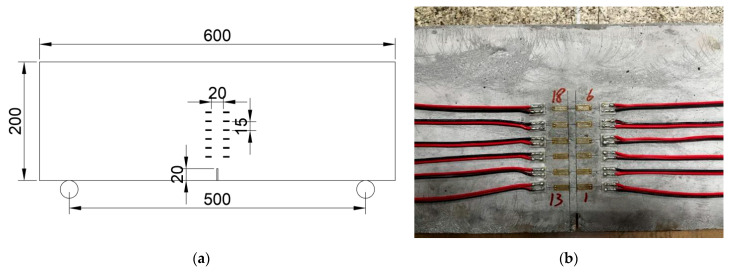
Installation of strain gauges along the height of the SENB specimen ahead of the prefabricated notch for crack size measurement: (**a**) Schematic illustration of strain gauge arrangement (dimensions in mm); (**b**) Photograph of strain gauges.

Because reduced strain measurement is usually a good indicator of concrete cracking, resistance strain gauges were affixed on the side surfaces of SENB specimens to determine the extended crack length during fatigue loading [12]. This strain reduction method proved effective, as the energy released during concrete cracking prevents further strain increase near the crack tip. The electrical resistance strain gauges had a gauge length of 10 mm, a width of 2 mm, and an electrical resistance of 120 Ω. Strain data were recorded using a UCS60B static data acquisition system at a sampling frequency of 0.28 s per channel. Strain gauges should be arranged in the vicinity of cracks, but should be avoided in the exact path of crack propagation to prevent tearing damage to the strain gauges. Since crack propagates in a zigzagging route in plain concrete, two rows of strain gauges were symmetrically installed on both sides of the prefabricated notch with a horizontal tip-to-tip distance of 20 mm. The vertical center-to-center distance between strain gauges is typically 15 mm and could be adjusted slightly to accommodate instrumentation needs. Since stable crack extension is of primary concern in fatigue crack growth rate tests, the strain gauges were arranged within the lower half of the specimen height, as shown in Figure 6.

**Figure 7:**
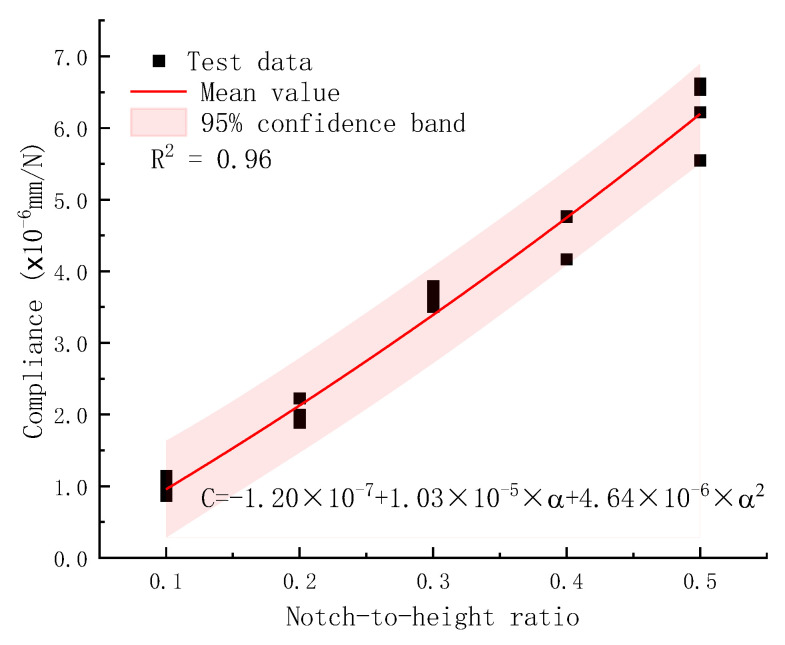
Calibrated elastic compliance versus notch-to-height ratio curve for plain concrete specimens. A unique notch-to-height ratio or relative crack size can be determined from one compliance value.

The relationship between compliance and notch-to-height ratio, used to determine crack length at different loading cycles in the fatigue crack growth rate tests, was derived from monotonic static loading tests in a previous study [37]. It was observed that the load *P* versus CMOD curve exhibited linear proportionality within the elastic region. The proportionality factor, *C* = CMOD/*P*, referred to as elastic compliance, depends solely on the notch-to-height ratio (or relative crack size), provided that the specimen geometry and concrete composition remain unchanged. The *P*–CMOD curve was established using 20 plain concrete specimens with different notch-to-height ratios, as reported in Reference [37]. The elastic compliance, defined as the slope of the linear portion of the *P*–CMOD curve for each specimen, was calculated and is presented in Figure 7 for various notch-to-height ratios. As notch depth increased, the remaining ligament length decreased, leading to a corresponding increase in compliance (the reciprocal of stiffness). These data points, together with the fitted regression curve (represented by the red line) and the 95% confidence band (pink region), are displayed in Figure 7. The compliance exhibited a parabolic relationship with the notch-to-height ratio, yielding an adjusted coefficient of determination of 0.96. The calibrated compliance versus notch-to-height ratio curve was subsequently employed to estimate crack length during fatigue crack propagation tests under cyclic loading.

### 2.5. Paris Law

The classical Paris’ law describes the relationship between fatigue crack growth rate and stress intensity factor range as in Equation (1):

(1) $$da/dN=C\cdot {\left(\Delta K\right)}^{n},$$

where d*a*/d*N* is fatigue crack growth rate; Δ*K* is stress intensity factor range defined as Δ*K* = *K*_max_ − *K*_min_, and *K*_max_ and *K*_min_ are the stress intensity factors associated with maximum and minimum fatigue loading; *C* and *n* are material constants.

A linear formula, Equation (2), is obtained by taking the logarithm on both sides of Equation (1).

(2) $$\mathrm{lg}\left(da/dN\right)=\mathrm{lg}C+n\mathrm{lg}\Delta K.$$

Therefore, material constants *C* and *n* can be obtained through regression analysis of data pairs of (lgΔ*K*, lg(d*a*/d*N*)). Stress intensity factor *K* at the crack tip of a three-point bending beam under a concentrated load *P* can be calculated by Equation (3).

(3) $$K=\frac{P}{B\sqrt{H}}f\left(\alpha \right).$$

While the specimen’s span-to-height ratio *S*/*H* is 2.5, the geometric function *f*(α) is

(4) $$f\left(\alpha \right)=\frac{6.647\alpha^{0.5}\left(1-2.5\alpha +4.49\alpha^{2}-3.98\alpha^{3}+1.33\alpha^{4}\right)}{{\left(1-\alpha \right)}^{3/2}},$$

where *B* is specimen width; *H* is specimen height; α is the relative crack size defined by the ratio between crack size and specimen height.

Once material constants *C* and *n* are known, fatigue crack propagation life extending a crack from initial size *a*_0_ to final size *a*_f_ in a structural component can be estimated through numerical integration of Equation (1), as shown in Equation (5).

(5) $$\int_{0}^{N}dN=\int_{a_{0}}^{a_{f}}\frac{da}{C{\left(\Delta K\right)}^{n}}.$$

### 2.6. Forman Equation

Experimental observations suggest that the stress ratio *R* may influence the fatigue crack growth rate under constant amplitude fatigue loading. If the fatigue load range Δ*P* is kept constant, an increased *R* tends to reduce fatigue life and increase d*a*/d*N*. Based on Paris Law, Forman Equation explicitly considers *R* and is expressed as in Equation (6):

(6) $$da/dN=\frac{C^{\prime }\cdot {\left(\Delta K\right)}^{n^{\prime }}}{(1-R)K_{\mathrm{IC}}-\Delta K}.$$

The rationale is that while *K*_max_ is approaching fracture toughness *K*_IC_, the crack propagates unstably and d*a*/d*N* is approaching infinity, that is, $\underset{\Delta K-(1-R)K_{\mathrm{IC}}\to 0}{\lim}da/dN=\infty$. During the fatigue test, *K*_max_ reaches its limiting value at the peak load of the last fatigue cycle, just before final abrupt failure. This onset of unstable crack extension (called fracture) corresponds to fracture toughness *K*_IC_, which can be obtained from a static fracture test.

Taking the logarithm on both sides of Equation (6) yields Equation (7):

(7) $$\mathrm{lg}\{(da/dN)[(1-R)K_{\mathrm{IC}}-\Delta K]\}=\mathrm{lg}C^{\prime }+n^{\prime }\mathrm{lg}\Delta K.$$

Material constants *C*′ and *n*′ in Equation (6) are different from those in Equation (1), and they can be obtained through regression analysis of data pairs of (lgΔ*K*, lg{(d*a*/d*N*)[(1 − *R*)*K*_IC_ − Δ*K*]}).

## 3. Results and Discussion

### 3.1. Material Characterization

The compressive strength of the C50 concrete mixture was determined through standard compression tests on nine cubes. The measured results and corresponding statistical parameters are summarized in Table 3. The average 28-day compressive strength of the batch was 65.4 MPa, confirming compliance with the strength specification for C50 commercial concrete. The coefficient of variation of 0.09 indicated acceptable consistency among specimens.

### 3.2. Static Peak Load

The fatigue behavior of ordinary concrete is extremely sensitive to the applied alternating fatigue load, which is predetermined as a certain proportion of its static load-carrying capacity. An accurate characterization of the static strength is therefore important, and a sufficient number of specimens is necessary, considering the large dispersion of concrete material properties. Therefore, a total of 12 SENB plain concrete specimens with a notch-to-height ratio of 0.1 were tested under static loading to obtain the average peak load. These specimens comprised six beams from series JZ-W-2 and six from series WJ. The series identifiers follow Chinese Pinyin acronyms: “JZ” denotes static loading (as opposed to stress-controlled fatigue loading, “S”), while “W” or “WJ” designates plain concrete. The number represents an initial notch depth of 2 cm. Please note that these 12 specimens were identical, except that strain gauges were installed on the JZ-W-2 series to measure crack initiation load in addition to the maximum load-carrying capacity. Other details of the fracture tests are provided in Reference [37]. The measured peak loads and corresponding statistical parameters from the fracture test are summarized in Table 4. The average peak load of 25.60 kN from all 12 specimens was used as static strength to determine the maximum and minimum fatigue loads.

### 3.3. Fatigue Failure Mode

**Figure 8:**
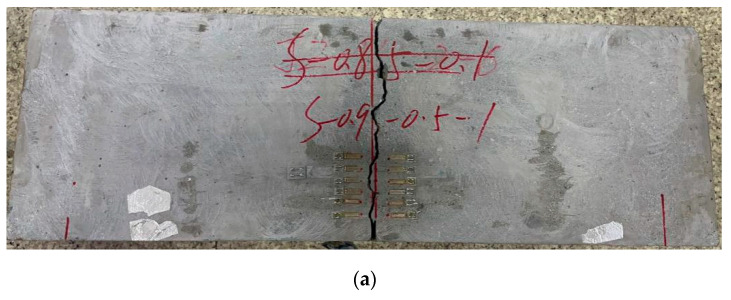

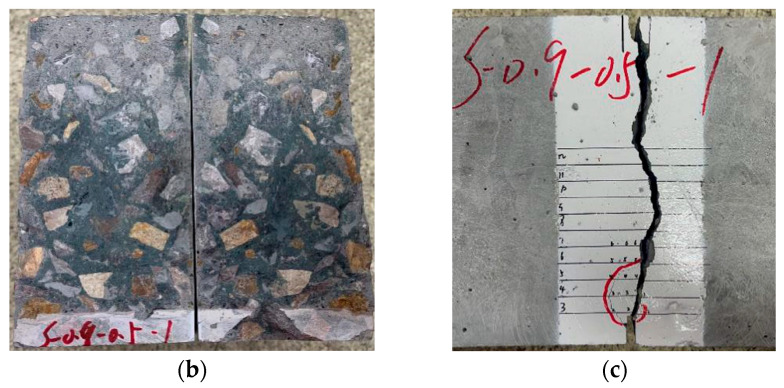
Fatigue crack propagation path and fracture surface of a typical fatigue fractured specimen: (**a**) Side view; (**b**) Cross-section view; (**c**) Crack propagation path.

The pattern of a typical specimen under fatigue loading is illustrated in Figure 8. As shown in Figure 8a, a fatigue crack originated at the prefabricated notch tip and propagated upward in a zigzag pattern. Once the crack reached approximately halfway through the specimen, it propagated rapidly, ultimately causing the specimen to fracture into two parts. The crack growth pattern under fatigue loading corresponded closely to the pattern observed under monotonic loading [37]. Final specimen failure was predominantly controlled by coarse aggregate fracture, as evidenced by the symmetric cross-sections in Figure 8b. Compared to monotonic loading, fatigue loading allowed for a more extended crack development period, facilitating observation of cracks on the specimen’s side surfaces. Multiple microcracks developed during fatigue loading, including oblique microcracks visible under a microscope in Figure 8c. Microcracks typically originated in the cement matrix and at the interface transitional zone (ITZ) between the matrix and coarse aggregates [38,39], and their coalescence into a single macrocrack eventually led to specimen fracture.

**Figure 9:**
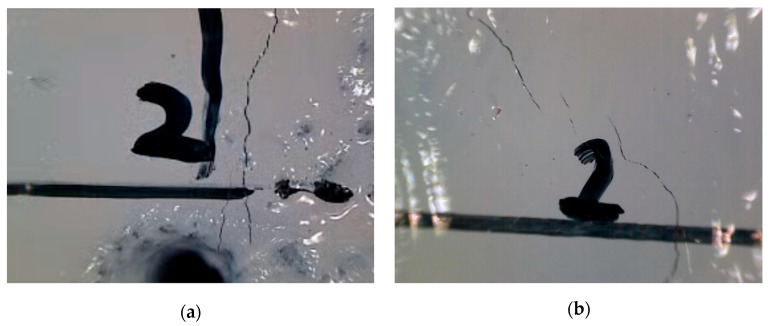

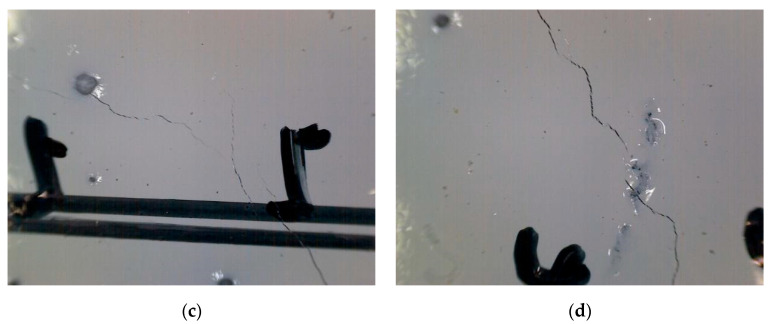
Observed microcracks using the electronic microscope during the fatigue crack growth rate test: (**a**) Multiple microcracks around the prefabricated notch; (**b**) Multiple microcracks at other locations; (**c**) Oblique microcrack; (**d**) Microcrack around surface void.

Observed with the electronic microscope, some unique features of microcracks during the fatigue test, compared to those due to monotonic loading, are further presented in Figure 9. First, multiple microcracks, as shown in Figure 9a, typically initiate from the prefabricated notch tip, which heavily relies on the geometric dimensions, fabrication process of the notch, and the distribution of coarse aggregate around the notch tip. Multiple microcracks were not only observed around the notch tip but also at other locations over the specimen height (Figure 9b). As the number of loading cycles increases, microcracks seemed to link up and propagate into a penetrated macrocrack. Figure 9c shows an oblique microcrack, resulting in a wayward macroscopic crack that does not grow perfectly vertically upward. Figure 9d shows the crack development around an initial imperfection on the specimen surface. The surface void acts as a stop hole that might temporarily stop a crack from growing. After several loading cycles, the crack bypasses the void and continues to grow at other locations around it.

Moreover, fatigue crack was observed to grow periodically, and crack propagation was no longer a continuous process. The crack grew suddenly after a certain number of fatigue cycles and remained stable for a while, until the next burst in crack size occurred. This pattern is consistent with the observations by other researchers [13]. The phenomenon can be explained by the heterogeneity of concrete materials and the tendency of crack growth to develop around the ITZ, providing an energy-efficient route and overcoming the resistance of coarse aggregates [40,41].

In short, the crack development during fatigue loading is more complicated than during monotonic loading. Although the major macroscopic crack leading to eventual fracture failure is similar, multiple microcracks are present during fatigue loading. These microcracks are closely related to the initial prefabricated notch and other initial surface imperfections. They originate from the specimen’s weak positions and grow locally as the number of cycles increases. These microcracks grow upwards, merge, and eventually form a major macrocrack that penetrates the complete thickness of the specimen. The growth of microcracks is not necessarily consistent with that of the major macrocrack. Moreover, these localized microcrack growths will non-negligibly affect the strain values from the strain gauges affixed to the specimen’s side surfaces.

### 3.4. Fatigue Life and Residual Capacity

A fatigue test was performed on 36 plain concrete SENB specimens with a notch-to-height ratio of 0.1. The applied maximum and minimum fatigue load, actual maximum stress level, actual stress ratio, and fatigue life are tabulated in Table 5. As explained in Section 2.3, a maximum number of loading cycles is specified. For those specimens that did not fail after the specified cycles, a monotonic static load was applied to break the specimen. The maximum load that the specimen can sustain, termed residual load carrying capacity, after a certain number of fatigue cycles, is listed in the last column of Table 5. Note that the applied fatigue load was calculated from the design maximum stress level, design stress ratio, and the specimen’s static strength determined from Table 4 of Section 3.2.

Even in carefully controlled experiments, Table 5 indicates that fatigue life varied by several orders of magnitude among specimens within the same test series. The fatigue life also varied across different test series. In general, the average fatigue life tends to increase with a decrease in maximum stress at a constant stress ratio, or with an increase in stress ratio at a constant maximum stress level. Both the maximum stress level and the stress ratio influenced the fatigue life of the SENB specimens.

The seven runout specimens showed significantly higher strength than that determined by the 12 specimens in the static load test. The measured residual capacity has an average value of 28.75 kN, a standard deviation of 1.13 kN, and a coefficient of variation of 0.04. The average residual strength is 12.3% higher than the static strength determined approximately one month earlier. The apparent contradiction demonstrates the large scatter inherent in concrete properties. It might also be due to a difference in the concrete’s age. As concrete ages, its strength increases. Therefore, it might be more appropriate to use age-adjusted static strength or to determine the static strength from specimens of the same age.

### 3.5. P–CMOD and CMOD–N Curves

**Figure 10:**
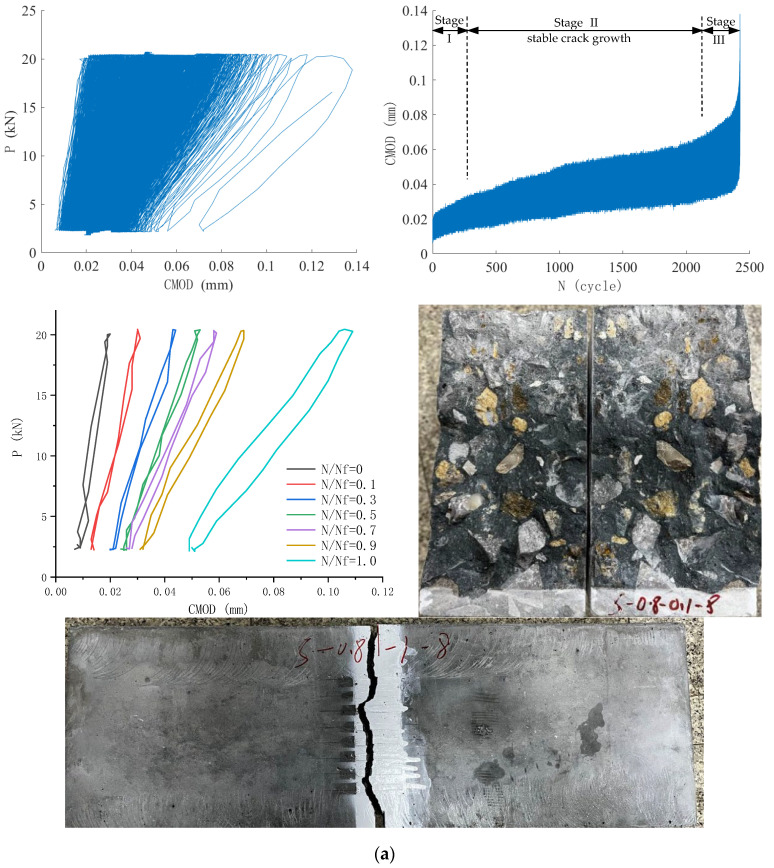

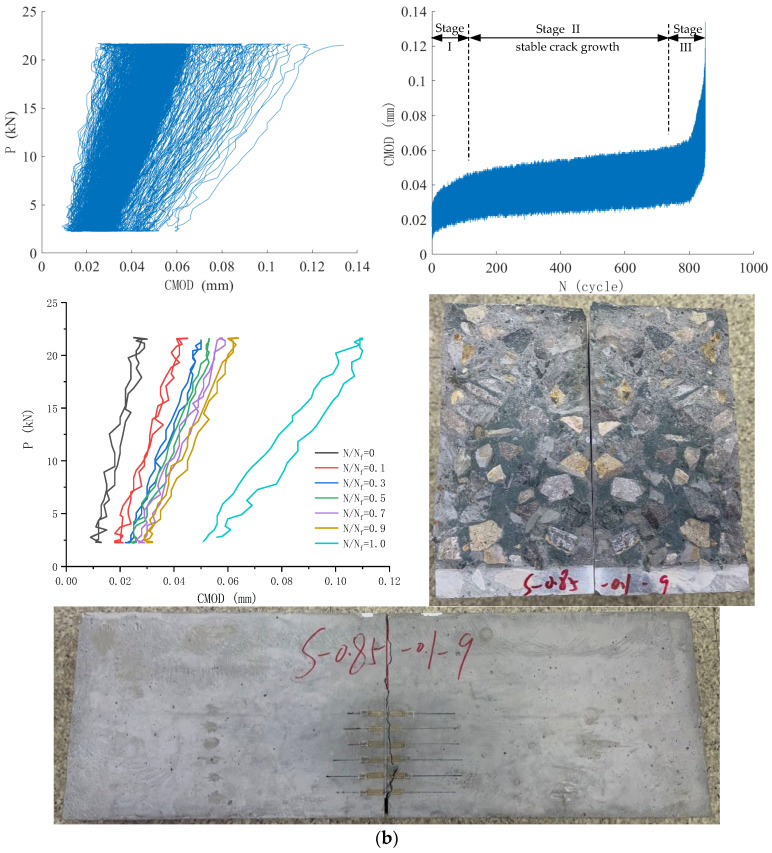
Typical load and CMOD results during fatigue test of SENB specimens: (**a**) Specimen S-0.80-0.1-8; (**b**) Specimen S-0.85-0.1-9.

Figure 10 shows load and crack mouth opening displacement (CMOD) results during the fatigue test of two typical SENB specimens, including the fatigue load versus CMOD curve and the CMOD versus fatigue cycles curve. *P*–CMOD curves at specified load cycle ratios *N*/*N*_f_ were also shown for clarity, where *N* is the applied load cycles and *N*_f_ is the specimen’s fatigue life. The cross-sectional view and side view of the fatigue-fractured specimen are also shown in Figure 10.

The fatigue test was conducted under load control, and the CMOD increased with the increase in fatigue loading cycles. Both CMOD at maximum and minimum fatigue loads increased with loading cycles and exhibited three stages of evolution: an initial rapid increase, a slow and stable increase, and a final rapid increase. Furthermore, the CMODrange—the difference between CMOD values at maximum and minimum loads— evolved similarly in a three-stage pattern. Therefore, under maximum stress levels of 0.80 and 0.85, fatigue progression of plain ordinary concrete can be divided into three distinctive stages: crack initiation, stable crack growth, and unstable crack growth till final fracture, where the peak CMOD reaches a critical value. The stable crack propagation stage evolves slowly and occupies about 60~80% of the specimen’s total fatigue lifetime, while crack initiation and unstable crack growth stages develop rapidly. The *P*–CMOD curves at specified load cycle ratios *N*/*N*_f_ demonstrate the decrease in stiffness or increase in compliance of the specimen, which lays the foundation for the compliance method to determine fatigue crack size in Section 3.6.3.

### 3.6. Crack Size Measurement

#### 3.6.1. Visual Observation

**Figure 11:**
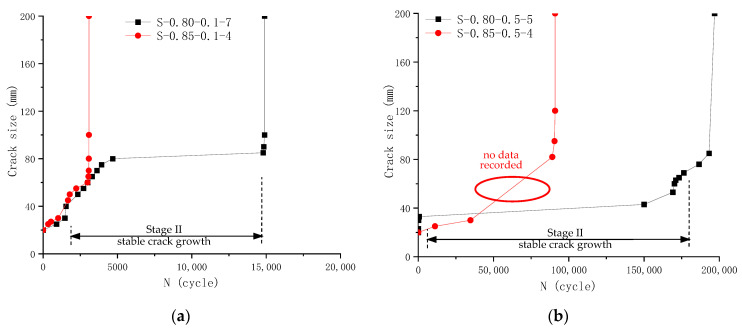
Fatigue crack propagation recorded by visual observation method: (**a**) Specimens S-0.80-0.1-7 and S-0.85-0.1-4; (**b**) Specimens S-0.80-0.5-5 and S-0.85-0.5-4.

Crack propagation was observed under an electron microscope during the fatigue test after spreading white lacquer on the specimen surface and adding grid lines with a black marker. The number of fatigue loading cycles for each observed crack growth was recorded. Visual observations were made on four specimens. The recorded crack sizes are tabulated in Table 6 and shown in Figure 11. Please note that although the microscope could help identify microcracks, the crack size was measured with a ruler, limiting precision to 1 mm.

Three distinctive stages of fatigue crack growth can be identified from visual observation data in Figure 11. It should be noted that in Figure 11b for specimen S-0.85-0.5-4, there is no intermediate data recorded from visual observation for cycles between 40,000 and 90,000, and the crack seems to change directly from 30 mm to 80 mm, as shown by the red ellipse. Therefore, this method can provide only limited value, and those that can provide more accurate and continuous monitoring of crack size need to be explored.

#### 3.6.2. Strain Gauges

**Figure 12:**
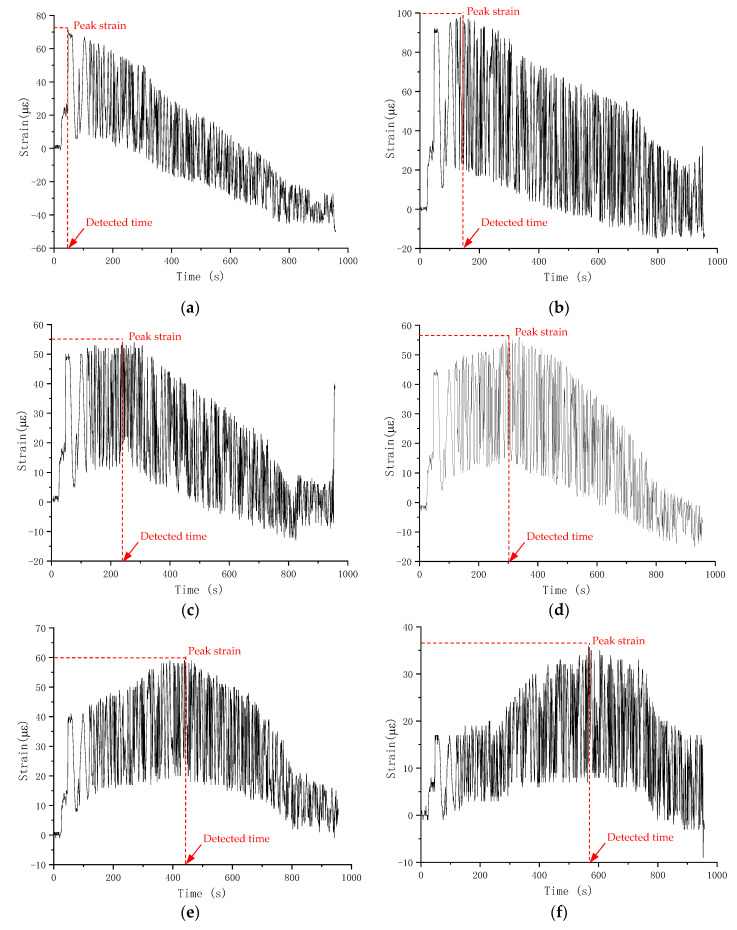

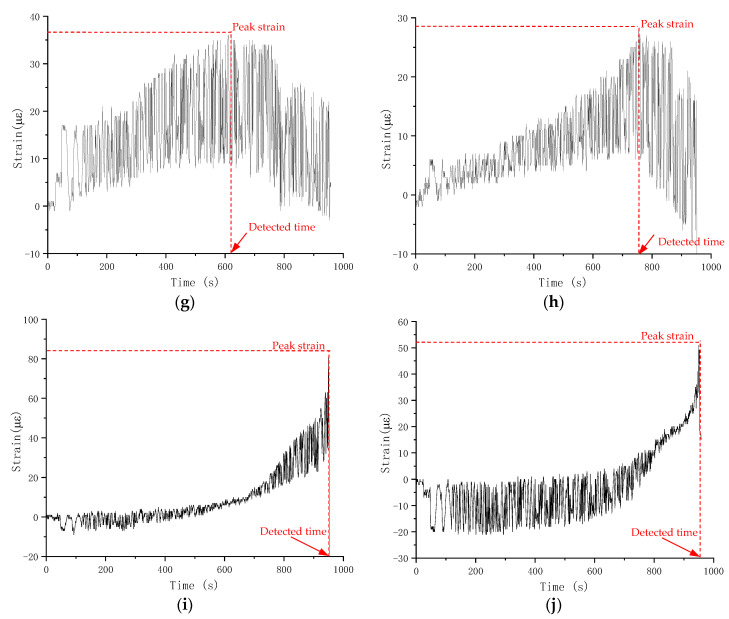
Measured strain value during fatigue test of Specimen S-0.80-0.1-1: (**a**) Gauge 1; (**b**) Gauge 2; (**c**) Gauge 3; (**d**) Gauge 4; (**e**) Gauge 5; (**f**) Gauge 6; (**g**) Gauge 7; (**h**) Gauge 8; (**i**) Gauge 9; (**j**) Gauge 10.

The strain gauge method is used to detect the progress of fatigue crack growth by measuring the reduced strain values resulting from energy release in the immediate region around the crack tip. However, strain values are affected by local surface conditions and microcrack development; stable strain data were collected from only a few fatigue test specimens. The recorded strain values for Specimen S-0.80-0.1-1 are shown in Figure 12 as an illustration.

Gauges 1 to 10 were affixed to the specimen’s side surface from bottom to top, as demonstrated similarly in Figure 6. Figure 12 illustrates that the strain values at different strain gauge positions, as local variables, are quite different and do not exhibit a clear trend. However, all strain readings tend to increase initially with time and then decrease after reaching a peak. The strain retraction is associated with energy release and crack propagation [12]. From Gauge 1 to 10, the time to peak strain increases, indicating that the crack grows gradually from bottom to top. The corresponding number of fatigue load cycles associated with strain retraction can be determined by synchronizing the time data from the strain gauge data acquisition system and that from the internal data recording system of the fatigue testing machine. The peak strain and associated number of fatigue cycles for strain gauges in Specimen S-0.80-0.1-1 are tabulated in Table 7.

**Figure 13:**
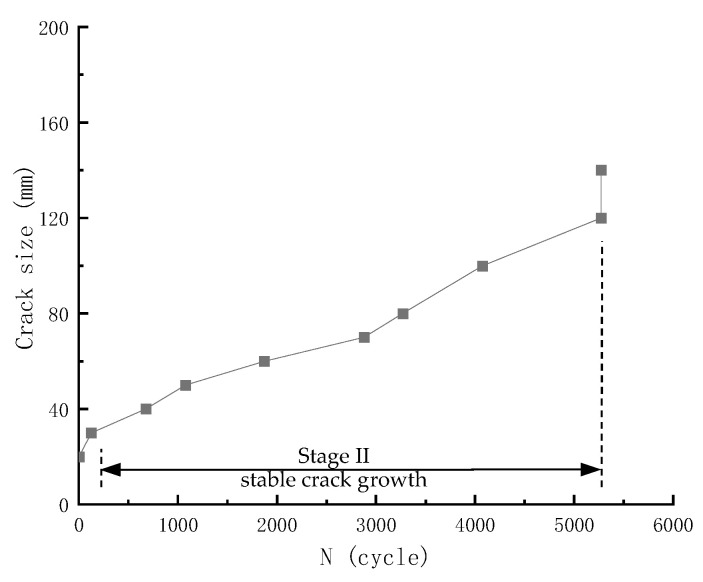
Fatigue crack propagation versus number of fatigue loading cycles of Specimen S-0.80-0.1-1, where crack size was determined from strain gauges affixed along the specimen height. Strain value reduces when the crack front reaches the position of the strain gauge.

Similarly, since the identified crack tip positions are restricted to locations where strain gauges were installed using a ruler, the measurement precision of crack size in this method is also 1 mm. The fatigue crack propagation size, deduced from strain gauge readings, as a function of the number of fatigue loading cycles, is shown in Figure 13.

Similarly, a three stage development of fatigue crack can be observed: strain retraction in Gauge 1 occurs immediately during the first loading cycle and specimen starts cracking rapidly; subsequent strain retraction in Gauges 3 to 8 indicate stable crack propagation of the second stage; the crack extends rapidly in final stage and Gauges 9 and 10 reached their peak value almost simultaneously, followed by strain gauge fracture and drastic change in strain value. As for fatigue life, the remaining fatigue cycles that the specimen can sustain when the fatigue crack extends to 10 cm long (i.e., indicated by strain retraction in Gauge 8) represent about 20.4% of the total fatigue life.

Since strain measurement is significantly influenced by local stress concentration and random microcrack development, deducing crack size from the strain retraction of successively arranged strain gauges is a complex process. Moreover, this method requires a large number of strain gauges to capture crack extension with sufficient accuracy. A more reliable and economical method to continuously monitor fatigue crack propagation is urgently needed and will be discussed in the next section.

#### 3.6.3. Compliance Method

Figure 7 in Section 2.4 shows a calibrated compliance curve from a previous static fracture test on geometrically and materially identical SENB specimens [37]. The effect of a fatigue crack on specimen compliance is assumed to be identical to that of a notch with the same length. A subcritical crack extension before static fracture is generally present in concrete, a quasi-brittle material. Such subcritical crack growth under fatigue loading is similarly accepted for concrete. The decrease in the specimen’s dynamic stiffness and increase in compliance are attributed to subcritical crack extension during fatigue loading [13]. Therefore, the calibrated compliance curve can be used for measuring fatigue crack size. It should be noted that the crack extension is an equivalent crack size considering the effect of numerous microcracks at the physical crack tip, not the physically observed macroscopic crack length.

**Figure 14:**
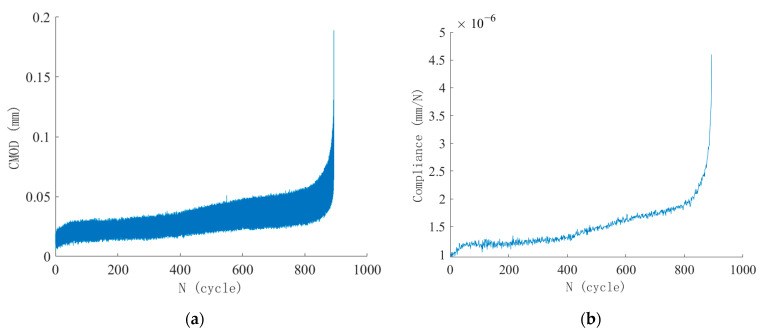
Evolution of crack mouth opening displacement (CMOD) and compliance during fatigue test of Specimen S-0.80-0.1-2: (**a**) CMOD–*N* curve; (**b**) Compliance–*N* (C–*N*) curve.

Fatigue crack size can be continuously deduced using the compliance method because the dynamic compliance under fatigue loading has a one-to-one correspondence with the dynamic crack size. Specimen S-0.80-0.1-2 was randomly selected to illustrate the application of the compliance method in crack size measurement. Based on the recorded CMOD–*N* curve in Figure 14a, the variation in compliance with the number of fatigue loading cycles was derived. The real-time elastic compliance for each loading cycle was determined from the slope of the linear elastic segment of the *P*–CMOD curve using linear regression analysis, and the corresponding values are presented in Figure 14b.

**Figure 15:**
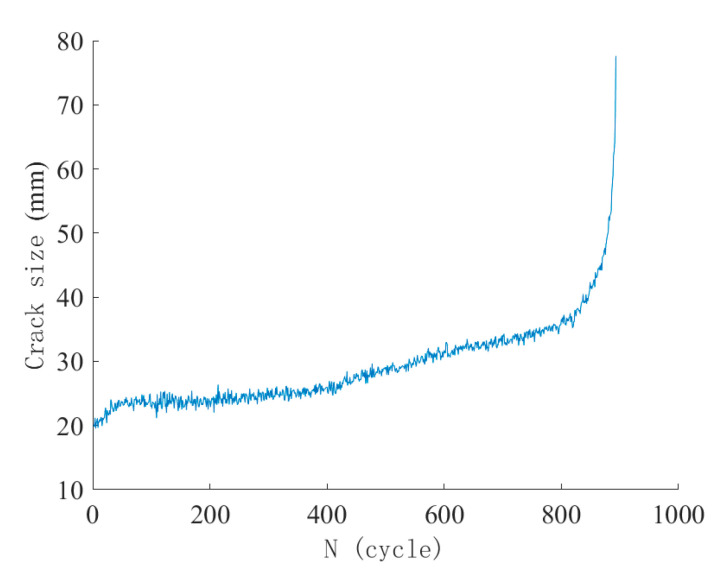
Crack size versus number of fatigue loading cycles for Specimen S-0.80-0.1-2.

Combining the C–*N* curve in Figure 14b and the C–α curve in Figure 7, the real-time relationship between crack size versus fatigue loading cycles can be established. The dynamic crack size at maximum fatigue load during each loading cycle is presented in Figure 15. The estimated equivalent crack size from the compliance method has been modified to ensure the initial crack size at the start of fatigue loading is 20 mm. It should be noted that although the precision of CMOD measurement is 0.001 mm, the precision of the derived crack size is about 1 mm, given the geometry and loading in this study.

#### 3.6.4. Comparison of Measurement Methods

**Figure 16:**
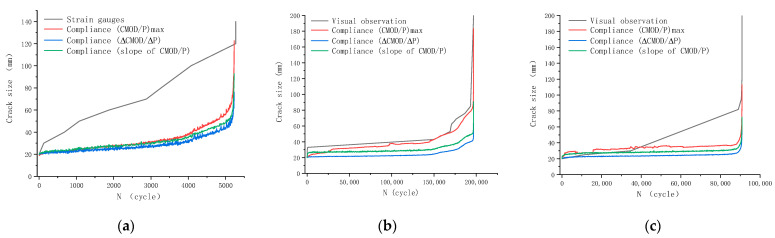
Comparison between crack size measurement methods: (**a**) Specimen S-0.80-0.1-1; (**b**) Specimen S-0.80-0.5-5; (**c**) Specimen S-0.85-0.5-4.

A comparison of crack size measurement methods was conducted on specimens with either visual observation or strain gauges, as shown in Figure 16. Table 8 summarizes quantitative deviations between these two methods and the compliance method.

Three methods of determining compliance in each cycle are also compared in Figure 16, namely, the red line for direct division of corresponding CMOD by peak load, the blue line of secant compliance calculated as CMOD divided by P, and the green line of tangent compliance calculated as the slope of the linear portion of the loading branch. By definition, the third method, tangent compliance, is the one adopted to obtain the compliance calibration curve in Figure 7 and the real-time elastic compliance curve in Figure 14. During most of fatigue life, these three methods derived comparable crack sizes. However, it should be pointed out that the compliance method tends to underestimate the crack size. This discrepancy arises because the fatigue crack width during stable propagation (in the order of 0.1 mm) is dramatically smaller than the prefabricated notch width (2 mm); the fatigue-cracked specimen has a lower compliance than the notched counterpart.

As demonstrated in Figure 16b,c (before 40,000 cycles, subsequent 50,000 cycles without recorded data) and Table 8, the crack size determined by the compliance method was comparable to that from visual observation, demonstrating the effectiveness of the method. The compliance method estimated a smaller crack size than visual observation during most of the fatigue life, which could be explained by the observation that the crack front in concrete SENB specimens is typically V- or U-shaped. However, Figure 16a showed an average relative difference of 113% between the strain gauge retraction method and the compliance method. This difference is probably due to strain gauge measurements being susceptible to local surface defects.

### 3.7. Crack Propagation by Paris Law

The crack measurement by the elastic compliance method (tangent compliance) in Figure 15 would be processed by the finite difference method for further analysis of the crack growth rate. Fatigue crack growth rate d*a*/d*N* can be approximated by finite difference Δ*a*/Δ*N*, and stress intensity factor range can be evaluated by Equations (3) and (4) with the relative crack length at the central point of differential intervals. To ensure CMOD’s measurement precision does not overshadow Δ*a*, Δ*N* was not kept constant. Δ*N* initially ranged in hundreds of cycles when the crack was small and gradually decreased to a single cycle near the end of the fatigue test. A MATLAB R2021a program developed by The MathWorks, Inc. (Natick, Massachusetts, USA) was implemented to facilitate the data processing using the classical 7-point polynomial fitting method [12].

**Figure 17:**
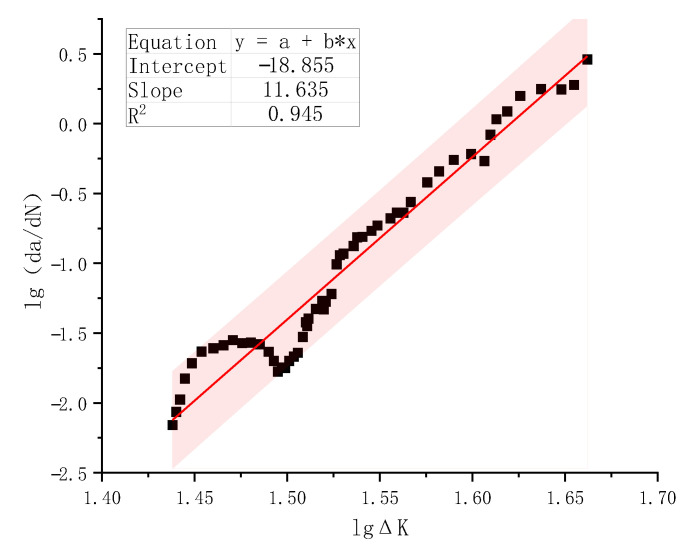
lg(d*a*/d*N*) versus lgΔ*K* test data points and regression analysis for Specimen S-0.80-0.1-2.

Based on the α–*N* curve in Figure 15, all data points of d*a*/d*N* versus Δ*K* were plotted on a logarithmic scale. Here, d*a*/d*N* is expressed in mm/cycle and Δ*K* in $MPa\cdot \sqrt{mm}$ (N-mm unit). These data points typically formed a linear scatter band. A linear regression analysis was performed to fit the data, and the resulting lg(d*a*/d*N*)−lgΔ*K* curve, including the 95% confidence interval (pink region), is presented in Figure 17.

With a coefficient of determination of 0.945, the Paris’ formula can describe the stable propagation of fatigue cracks reasonably well. With the fitted material constants *n* = 11.635 and lg*C* = −18.855, the average fatigue crack growth rate of ordinary plain concrete C50 obtained from specimen S-0.80-0.1-2 can be expressed as in Equation (8).

(8) $$da/dN=1.40\times 10^{-19}{\left(\Delta K\right)}^{11.635}.$$

**Figure 18:**
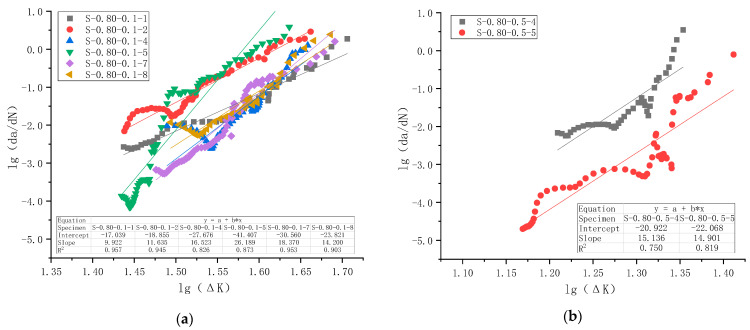

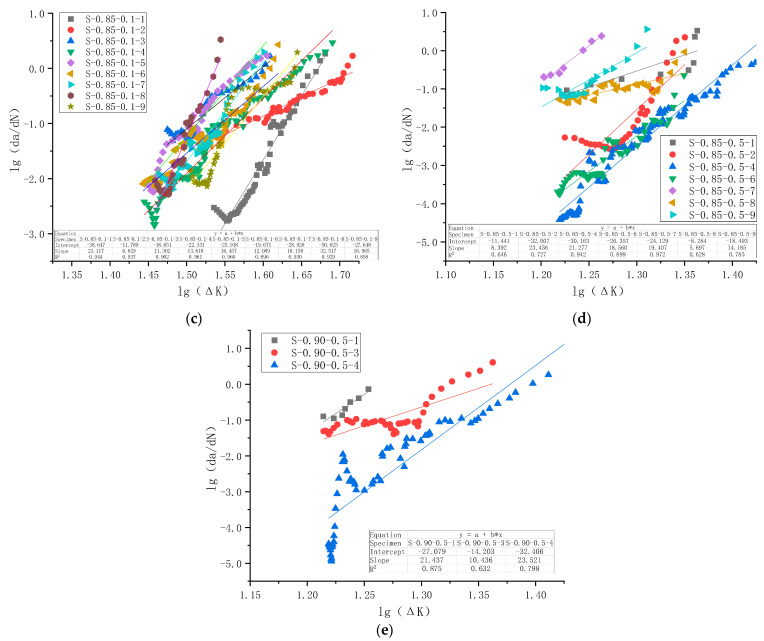
lg(d*a*/d*N*) versus lgΔ*K* test data points and regression analysis for all specimens in the fatigue crack growth rate test: (**a**) Series C1 with *S*_max_ = 0.80 and *R* = 0.1; (**b**) Series C2 with *S*_max_ = 0.80 and *R* = 0.5; (**c**) Series C3 with *S*_max_ = 0.85 and *R* = 0.1; (**d**) Series C4 with *S*_max_ = 0.85 and *R* = 0.5; (**e**) Series C5 with *S*_max_ = 0.90 and *R* = 0.5.

The same procedure was applied to all 29 specimens that failed under fatigue loading. Specimens S-0.80-0.1-9 and S-0.80-0.5-2 were excluded from analysis because their fatigue life is too short for such an analysis. Altogether 27 specimens were curve-fitted based on the compliance method and Paris Law, and the results are shown in Figure 18 for all five series.

Regression analysis results and the average values of slope *n* and intercept lg*C* for each series of specimens and their combinations are summarized in Table 9.

It is observed from Figure 18 that the goodness of fitting of each specimen is reasonably good, with the minimum value of the coefficient of determination R^2^ as 0.63. However, there is significant scatter between specimens in the same series, as shown in Figure 18. Therefore, when these specimens were combined for regression analysis, the coefficient of determination could be low. As shown in Table 9, the maximum value of the coefficient of determination R^2^ for five series (Cases 1–5) is 0.63, and R^2^ for C4 is merely 0.25. This discrepancy is caused, on the one hand, by specimen-to-specimen scatter, and on the other hand, by limited data points for some specimens due to limitations in crack measurement. It is unclear to what extent this would undermine the overall applicability of Paris Law to plain concrete. However, analysis of R^2^ across different series combinations revealed several interesting observations. R^2^ of Cases 6 and 7, when a series of specimens is grouped for the same maximum stress levels, is smaller than that of the original series. While the R^2^ of Cases 8 and 9, when a series of specimens is grouped for the same stress ratio, lies between those of separate series. Following the same logic, when all specimens are combined, R^2^ in Case 10 is small. Since the small R^2^ in series C4 might have an effect, C4 was excluded from the analysis in Cases 11 and 12, and the resultant R^2^ has improved compared with Cases 9 and 10.

Algebraic mean values of the slope *n* and intercept lg*C* for each specimen series (or combinations of series) are presented in the fourth-to-last and second-to-last columns of Table 9. The table indicates that the values of *n* and lg*C* obtained via this method differ markedly from those derived from regression analysis, with the discrepancy exceeding acceptable limits in 5 of the 12 cases considered. The average method, treating each specimen in a series equally, yields more consistent results for *n* and lg*C* when processing data from multiple specimens. In contrast, regression-based constants fluctuate and are not always correct. In regression analysis, each data point is treated equally. However, each specimen does not necessarily have the same number of data points (see Figure 18e, for example, for the apparent difference). These two methods produce identical results if the number of data points is kept equal for each specimen. Since the average method is simple to use, logically correct, and has been reported in the literature [13], the algebraic average values of slope *n* and intercept lg*C* are recommended for subsequent analysis.

**Figure 19:**
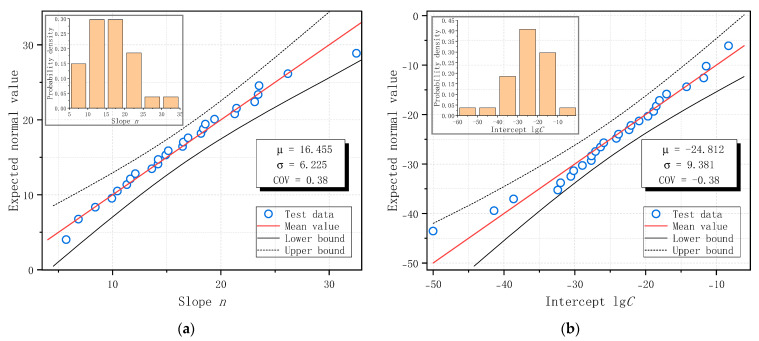
Normal distribution of fatigue crack growth constants of C50 ordinary plain concrete based on Paris Law: (**a**) Slope *n*; (**b**) Intercept lg*C*.

The fatigue crack growth constants obtained from each specimen were arranged in ascending order, and the Normal probability distribution plots for the slope *n* and intercept lg*C* are presented in Figure 19. The distribution parameters, including the mean and coefficient of variation (COV), were estimated from the dataset and are displayed in Figure 19, along with a 95% confidence interval. All test data for *n* and lg*C* fell within the 95% confidence band and closely followed the linear Normal distribution trend. Therefore, the measured material constants based on Paris Law were found to follow a Normal probability distribution in this study. The last third and first columns of Table 9 show the COV values of the slope *n* and intercept lg*C* for each specimen series (or combinations of series).

### 3.8. Crack Propagation by Forman Equation

**Figure 20:**
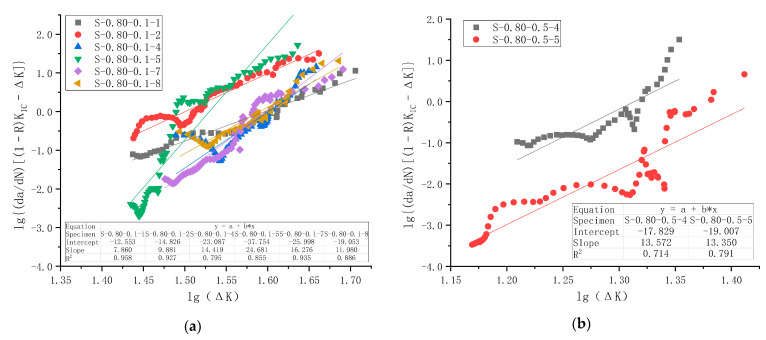

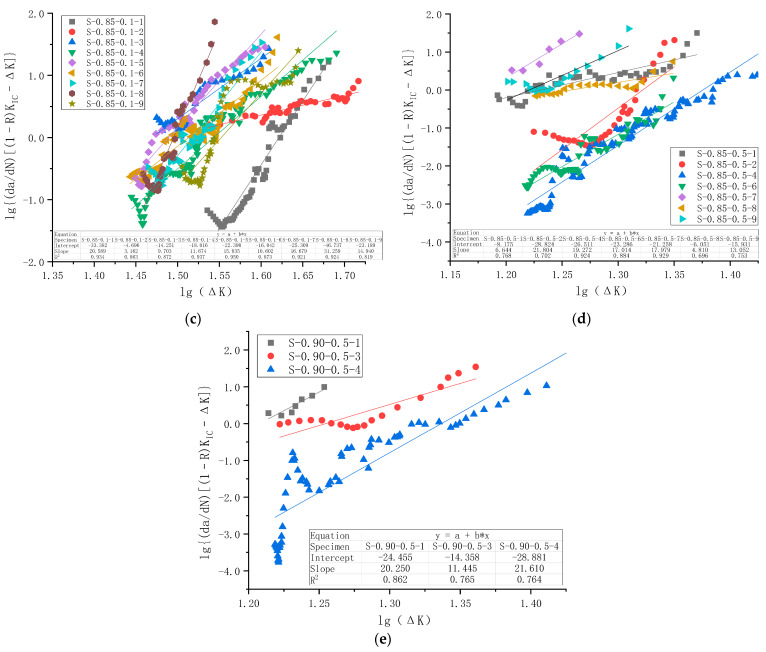
lg{(d*a*/d*N*)[(1 − *R*)*K*_IC_ − Δ*K*]} versus lgΔ*K* test data points and regression analysis for all specimens of the fatigue crack growth rate test: (**a**) Series C1 with *S*_max_ = 0.80 and *R* = 0.1; (**b**) Series C2 with *S*_max_ = 0.80 and *R* = 0.5; (**c**) Series C3 with *S*_max_ = 0.85 and *R* = 0.1; (**d**) Series C4 with *S*_max_ = 0.85 and *R* = 0.5; (**e**) Series C5 with *S*_max_ = 0.90 and *R* = 0.5.

Regression analysis was similarly conducted following Equation (7). Forman Equation explicitly considered stress ratio *R*, and the results are shown in Figure 20 for all five series of 27 specimens with *R* of 0.1 and 0.5. The average values of slope *n*′ and intercept lg*C*′ for each series of specimens and combinations of series are summarized in Table 10. Fracture toughness *K*_IC_ of C50 concrete was taken as a constant of 2.0 $MPa\cdot \sqrt{m}$ from a previous study on the same material [37]. Since the unit of *K* is in $MPa\cdot \sqrt{mm}$ (N-mm unit) in this study, this *K*_IC_ value is converted to 63.25 $MPa\cdot \sqrt{mm}$. The unit of d*a*/d*N* is still mm/cycle as before.

**Figure 21:**
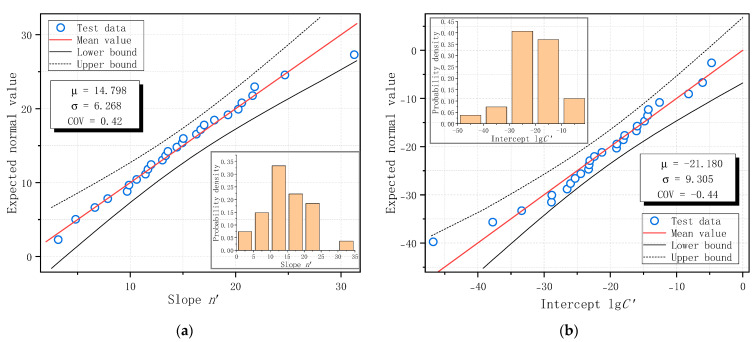
Normal distribution of fatigue crack growth constants of C50 ordinary plain concrete based on Forman Equation: (**a**) Slope *n*′; (**b**) Intercept lg*C*′.

The calculated material constants of all specimens were arranged in ascending order to obtain the probability plot. The corresponding Normal distribution plots, together with the estimated distribution parameters and 95% confidence bounds, are presented in Figure 21. The results indicate that all test data fall within the 95% confidence band and align well with the theoretical Normal distribution line. This finding confirms that the probability distribution of the slope and intercept derived from Forman Equation follows a Normal distribution. The COV values of the slope *n*′ and intercept lg*C*′ for all specimens and their combined series are summarized in Table 10.

A comparison of material parameters from Forman Equation and Paris Law indicates a decreased slope value and an increased intercept value for all cases, as shown in Table 9 and Table 10. This difference is because the vertical d*a*/d*N* is modified by multiplying [(1 − *R*)*K*_IC_ − Δ*K*] = [(1 − *R*)(*K*_IC_ − *K*_max_)], which is always positive and decreases with increasing Δ*K.* For the stable crack propagation stage of *K*_max_ lying between 30% and 80% of *K*_IC_ [8], such a modifier for the case of *R* = 0.5 is 22 $MPa\cdot \sqrt{mm}$ for small Δ*K* and approaching 6 $MPa\cdot \sqrt{mm}$ for large Δ*K* towards the end of the fatigue test.

Therefore, this study concludes that both Paris Law and Forman Equation can roughly describe the mode I fatigue crack behavior of ordinary plain concrete under flexural tension. It should be noted that enormous discrepancies exist in the test results, with the majority of COV lying in ±(0.30~0.40).

### 3.9. Comparison with the Literature

Fatigue crack growth rate data obtained from SENB specimens tested under three-point bending with ordinary concrete of strength grades between 30 and 60 MPa were collected from the literature. These data, together with the results of this study, are summarized in Table 11 for direct comparison.

A direct comparison of the obtained crack growth constants with those in the literature can be confusing and complicated, as the units of crack growth rate and stress intensity factor are sometimes missing or not explicitly stated. The crack size could be in m, cm, or mm, and the stress intensity factor could be in $MPa\cdot \sqrt{m}$, $MPa\cdot \sqrt{cm}$, or $MPa\cdot \sqrt{mm}$, let alone imperial units. Even for the same datasets, if d*a*/d*N* or Δ*K* is expressed in different units, the fitted material constants *C* and *n* from regression analysis are different. If the units adopted were specified, at least the obtained parameters can be converted (as has been carried out in Table 11). In the worst case, the units were missing. Therefore, it is strongly recommended to use a unified unit in reporting data, and the SI unit is recommended, that is, N for load or mm for length.

**Figure 22:**
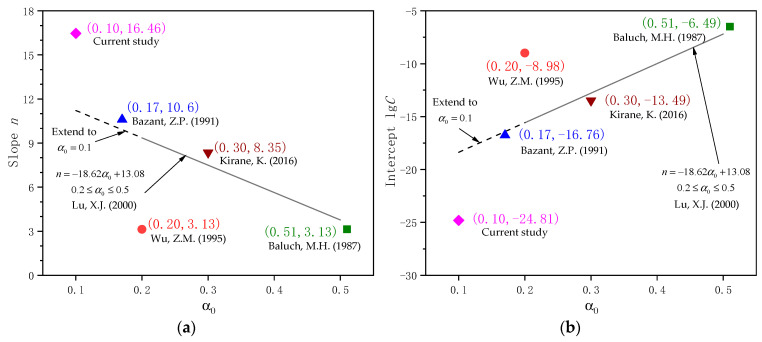
Comparison of calculated slope and intercept from this study with data from the literature (Wu, Z.M. (1995) [5], Baluch, M.H. (1987) [8], Bazant, Z.P. (1991) [9], Kirane, K. (2016) [11], and Lu, X.J. (2000) [13] respectively) with respect to the initial notch -to-height ratio α_0_. (**a**) Slope *n*; (**b**) Intercept lg*C*.

An initial observation of the reported Paris Law parameters across various references appears contradictory: the slope values ranged from 3 to 17, and the intercept values ranged from −25 to −6. In reference [13], crack propagation parameters were found to be dependent on initial notch size and were derived from α_0_ between 0.2 and 0.5. The corresponding slope values are 9.36, 7.49, and 3.77, and the intercept values are −15.57, −12.78, and −7.20 for α_0_ = 0.2, 0.3, and 0.5, respectively. If these expressions are assumed to be valid while extending below α_0_ of 0.2, the corresponding slope values are 11.22 and 9.91, and the intercept values are −18.37 and −16.41 for α_0_ = 0.1 and 0.17, respectively. These values are shown in Figure 22, along with the results from respective references.

As shown in Figure 22, both slope and intercept in References [8,9,11,13] correlate with each other quite well. This dependence on initial notch size might be explained as follows: at higher α_0_, the initial *K*_max_ and Δ*K* are relatively large, and the stable crack growth can be maintained only with a smaller crack growth rate, resulting in a smaller slope and a larger intercept. Even though Reference [8] has a much higher specimen span-to-depth ratio, it did not seem to affect the results. Although the trend is consistent with that reported in the literature, there are relatively large differences between Reference [8] and the current study, indicating that studies on fatigue crack growth properties remain inconclusive. With a larger slope (16.46 versus 11.22, 46.7% higher) and smaller *K*_IC_ (63.25 $MPa\cdot \sqrt{mm}$ versus 74.63 $MPa\cdot \sqrt{mm}$, 15.2% lower), fatigue cracks of the C50 concrete in this study tend to propagate faster than those in the literature [13], raising concerns of a higher risk of brittle failure in the studied commercial concrete.

### 3.10. Research Limitations and Future Work

Experiments show that fatigue cracks in concrete are torturous due to macroscopic material nonuniformity and the presence of initial imperfections. Even in the simple case of a three-point-bending concrete beam with a prefabricated notch, the fatigue crack is far from straight compared to its steel counterpart. Therefore, it is even questionable to define crack size or length from visual observation. The indirect measurement of crack size by the compliance method is also far from perfect, as prefabricated notch width is much broader than the actual crack width and aggregate locking effect in cracked concrete, resulting in a much smaller crack size derived from the compliance calibration curve. On the other hand, more advanced, accurate crack measurement techniques are a prerequisite to obtain reliable fatigue crack propagation properties of concrete, especially to overcome the inherent large scatter in concrete material properties. The crack-detection methods, explored in the literature for acoustic emission (AE) [14,22,23,26,29], digital image correlation (DIC) [14,20,24,30,31], and deep learning convolutional neural networks [42,43], should be applied in the future.

The definition of crack length remains challenging for multiple cracks, as shown in Figure 9. Fortunately, the fractal theory provides an effective means for quantitatively describing chaotic phenomena using mathematical language. The number and density of cracks could be described using a fractional dimension. The variation in the fractional dimension with the number of cycles can be inferred from experimental observations. Recent efforts have focused on determining the constants *C* and *n* in Paris Law based on the theory of energy equivalence between damage and cracks. Analytical models were proposed based on dimensional analysis, fractal geometry, and incomplete self-similarity [44]. They tried to explain the physical meaning of Paris Law constants and the inherent size effect law in quasi-brittle materials [45,46]. The influence of global energy, energy release rate, and fatigue fracture energy on fatigue crack propagation has been investigated by various scholars [47,48]. Although complex and still under development, these models are promising and warrant further exploration to understand the underlying crack propagation mechanism.

Since fracture toughness is size dependent, fatigue crack growth rate may also depend on specimen size, as indicated by Forman Equation. The size effect on fatigue crack propagation properties—an important issue—should be systematically studied. Moreover, as discussed in the previous section, the dependence of fatigue crack propagation parameters on initial notch size also needs further investigation.

The differences in crack development between fatigue and monotonic loading remain purely observational. In the future, fractographic examination and SEM image analysis should be conducted to provide quantitative evidence and explain the underlying mechanisms, such as aggregate interlock, ITZ weakening, or cumulative damage under cyclic loading.

One practical implication of the findings on fatigue crack propagation properties is the evaluation of fatigue performance in existing bridges and concrete structures, as implied by Equation (5). Since cracking is almost unavoidable in concrete structures where non-negligible fatigue tensile stress exists, further investigation into the influence of fatigue crack propagation on the durability of concrete structures, such as concrete’s impermeability and carbonation depth, transport channel of erosive ions, and hence corrosion resistance of reinforcing bars, is also warranted.

## 4. Conclusions

A three-point bending test was performed on 48 C50 plain concrete single-edge notched beam (SENB) specimens with a notch-to-height ratio α_0_ of 0.1. Among them, twelve specimens were subjected to static loading to determine their ultimate load-carrying capacity, and 36 were tested under dynamic fatigue loading for crack growth properties of ordinary concrete. The maximum fatigue load was taken as 0.80, 0.85, and 0.90 times the peak load from the static fracture test (maximum stress level *S*_max_), and stress ratios *R* of 0.1 and 0.5 were considered. The fatigue specimens were tested under stress control and were grouped into five series. Fatigue failure pattern, fatigue life, and the variation in crack mouth opening displacement (CMOD) were reported. Three methods for determining fatigue crack size—visual observation, strain gauges, and the compliance method—were explored, and the compliance method was selected for this study. Test data was further processed to obtain fatigue crack propagation properties according to Paris’ law and Forman’s equation. The obtained results were evaluated against existing literature, leading to the following conclusions:

(1) Using a calibrated compliance curve established in a previous study, the compliance method effectively monitored the fatigue crack size in real time and accurately identified the three stages of fatigue crack propagation (approximately 10%, 80%, and 10% of total fatigue life for stages I, II, and III, respectively). Elastic or tangent compliance, determined from the slope of the linear portion of the loading branch of the CMOD–*P* curve, is recommended for analysis. However, the derived crack size as an equivalent value is consistently smaller than that from visual observation, due to a convexed crack front and a thinner width of crack compared to the prefabricated notch.

(2) Fatigue crack growth rate test data d*a*/d*N* of an individual specimen can be related to stress intensity factor range Δ*K* as described by Paris Law reasonably well, with a minimum value of the coefficient of determination R^2^ as 0.63. However, when all specimens from one series were combined, the goodness of fitting from the regression analysis is low due to large scatter between specimens and limitations in crack size measurement. Fatigue crack growth constants follow a Normal distribution, and algebraic average values of slope and intercept are recommended for practical applications.

(3) Both the Paris Law and Forman Equation can describe mode I fatigue crack behavior of concrete loaded under flexural tension reasonably well. The slope values from Forman Equation are always smaller than those from Paris Law, and vice versa for the intercept value, for ordinary plain concrete with fracture toughness *K*_IC_ larger than 35 $MPa\cdot \sqrt{mm}$ and tested with *R* ≤ 0.5.

(4) With N-mm units, the average slope value of C50 concrete based on 27 fatigue failed SENB specimens from this study, according to Paris Law, is 16.46 with a coefficient of variation of 0.38, and the average intercept value is −24.81 with a coefficient of variation of −0.38. The average value and coefficient of variation in slope and intercept of ordinary plain concrete, according to Forman Equation, are 14.80 and 0.42 and −21.18 and −0.44, respectively.

(5) Comparison with existing literature confirmed that the crack growth constants in Paris Law depend on the notch-to-height ratio α_0_. As α_0_ decreases, the slope value increases and the intercept value decreases. C50 concrete in this study shows a 46.7% larger slope (16.46 versus 11.22) than those in the literature. Since fatigue crack growth is affected by ductility, higher-strength ordinary concrete tends to propagate faster than lower-strength concrete, and its ability to resist stable crack propagation should be of concern.

The engineering implications of the findings in this study on the fatigue crack propagation properties of C50 concrete include fatigue performance evaluation of existing bridges and fatigue design of bridge concrete. However, given the limitations of the crack size measurement method and the limited number of specimens from a single mixture and geometry tested under constant amplitude loading, more research is definitely necessary to support meaningful design code recommendations.

## Figures and Tables

**Table 1:** Detailed mix parameters of the commercial C50 concrete employed in this study [37].

Parameters	Cement	Fine Aggregate	Coarse Aggregate	Water	Additive Agent	Mineral Powder	Fly Ash
Properties	P.O 42.5	Medium sand	Crushed, 5~25 mm	–	STD-PCS	S95	Grade II, Class F
Source	Tianrui Cement Group	Zunhua, Hebei	Sanhe, Hebei	–	Tianjin Steady Industrial Development	Tianjin Iron & Steel Group	Tianjin Hydrus Group
Mass per m^3^ of concrete (kg/m^3^)	347	670	1048	160	9.20	69	46
Mix ratio	1	1.93	3.02	0.46	0.03	0.20	0.13

**Table 2:** Details of single-edge notched beam (SENB) specimens and fatigue loading in the three-point bending fatigue crack growth rate test.

Series	Specimen	Description	Maximum Stress Level *S*_max_	Stress Ratio *R*	Number of Specimens
C1	S-0.80-0.1-i	Fatigue crack growth rate test	0.80	0.1	9
C2	S-0.80-0.5-i	Fatigue crack growth rate test	0.80	0.5	5
C3	S-0.85-0.1-i	Fatigue crack growth rate test	0.85	0.1	9
C4	S-0.85-0.5-i	Fatigue crack growth rate test	0.85	0.5	9
C5	S-0.90-0.5-i	Fatigue crack growth rate test	0.90	0.5	4

**Table 3:** Measured 28-day compressive strength of cube specimens and corresponding statistical parameters [37].

Group	Measured Compressive Strength (MPa)			Statistical Parameters		
	Cube 1	Cube 2	Cube 3	Mean Value (MPa)	Standard Deviation (MPa)	Coefficient of Variation
1	69.8	64.8	56.4	63.67	6.77	0.11
2	73.4	61.5	72.5	69.13	6.63	0.10
3	63.9	65.9	60.7	63.50	2.62	0.04

**Table 4:** Measured static peak loads and statistical parameters [37]. The average value of 12 SENB specimens is used to determine the maximum and minimum fatigue load.

Series	Measured Peak Load (kN)						Statistical Parameters		
	Beam 1	Beam 2	Beam 3	Beam 4	Beam 5	Beam 6	Mean Value (kN)	Standard Deviation (kN)	Coefficient of Variation
JZ-W-2	24.81	24.06	23.12	25.59	24.04	24.34	24.33	0.83	0.03
WJ	21.66	27.11	28.77	28.31	33.39	21.99	26.87	4.45	0.17
Combined	24.81 21.66	24.06 27.11	23.12 28.77	25.59 28.31	24.04 33.39	24.34 21.99	25.60	3.33	0.13

**Table 5:** Applied fatigue load, fatigue life, and residual capacity of fatigue test specimens.

Series	Specimen	Minimum Load (kN)	Maximum Load (kN)	Actual Maximum Stress Level	Actual Stress Ratio	Fatigue Life (Cycle)	Residual Capacity (kN)
C1	S-0.80-0.1-1	2.345	20.405	0.797	0.115	5243	N.A. ^1^
	S-0.80-0.1-2	2.282	20.490	0.800	0.111	893	N.A. ^1^
	S-0.80-0.1-3	2.135	20.550	0.803	0.104	100,000 ^2^	27.700
	S-0.80-0.1-4	2.147	20.380	0.796	0.105	3541	N.A. ^1^
	S-0.80-0.1-5	2.167	20.333	0.794	0.107	43,064	N.A. ^1^
	S-0.80-0.1-6	2.037	20.465	0.799	0.100	150,000 ^2^	28.200
	S-0.80-0.1-7	2.030	20.468	0.800	0.099	14,898	N.A. ^1^
	S-0.80-0.1-8	2.085	20.417	0.798	0.102	2424	N.A. ^1^
	S-0.80-0.1-9	2.082	20.413	0.797	0.102	3	N.A. ^1^
C2	S-0.80-0.5-1	10.292	20.418	0.798	0.504	100,000 ^2^	29.160
	S-0.80-0.5-2	10.328	20.357	0.795	0.507	18	N.A. ^1^
	S-0.80-0.5-3	10.328	20.357	0.795	0.507	120,000 ^2^	27.700
	S-0.80-0.5-4	10.305	20.398	0.797	0.505	2124	N.A. ^1^
	S-0.80-0.5-5	10.260	20.432	0.798	0.502	196,835	N.A. ^1^
C3	S-0.85-0.1-1	2.307	21.602	0.844	0.107	5095	N.A. ^1^
	S-0.85-0.1-2	2.263	21.643	0.845	0.105	382	N.A. ^1^
	S-0.85-0.1-3	2.255	21.662	0.846	0.104	178	N.A. ^1^
	S-0.85-0.1-4	2.103	20.408	0.797	0.103	3082	N.A. ^1^
	S-0.85-0.1-5	2.140	20.387	0.796	0.105	733	N.A. ^1^
	S-0.85-0.1-6	2.237	21.670	0.846	0.103	997	N.A. ^1^
	S-0.85-0.1-7	2.237	21.667	0.846	0.103	882	N.A. ^1^
	S-0.85-0.1-8	2.252	21.643	0.845	0.104	831	N.A. ^1^
	S-0.85-0.1-9	2.232	21.610	0.844	0.103	850	N.A. ^1^
C4	S-0.85-0.5-1	10.920	21.673	0.847	0.504	309	N.A. ^1^
	S-0.85-0.5-2	10.943	21.663	0.846	0.505	4343	N.A. ^1^
	S-0.85-0.5-3	10.915	21.693	0.847	0.503	80,000 ^2^	30.977
	S-0.85-0.5-4	10.902	21.713	0.848	0.502	90,911	N.A. ^1^
	S-0.85-0.5-5	10.943	21.668	0.846	0.505	200,000 ^2^	28.840
	S-0.85-0.5-6	10.932	21.699	0.848	0.504	24,439	N.A. ^1^
	S-0.85-0.5-7	10.942	21.668	0.846	0.505	46	N.A. ^1^
	S-0.85-0.5-8	10.938	21.667	0.846	0.505	276	N.A. ^1^
	S-0.85-0.5-9	10.920	21.678	0.847	0.504	162	N.A. ^1^
C5	S-0.90-0.5-1	11.582	22.947	0.896	0.505	52	N.A. ^1^
	S-0.90-0.5-2	11.572	22.952	0.897	0.504	100,000 ^2^	28.643
	S-0.90-0.5-3	11.572	22.922	0.895	0.505	224	N.A. ^1^
	S-0.90-0.5-4	11.570	22.933	0.896	0.505	40,203	N.A. ^1^

^1^ Residual capacity was not measured because the specimen failed during the fatigue test. ^2^ The specimen did not fail after the specified number of fatigue cycles.

**Table 6:** Fatigue crack extension detected by visual observation of the electronic microscope on selected specimens.

S-0.80-0.1-7		S-0.80-0.5-5		S-0.85-0.1-4		S-0.85-0.5-4	
Loading Cycle	Crack Size (mm)	Loading Cycle	Crack Size (mm)	Loading Cycle	Crack Size (mm)	Loading Cycle	Crack Size (mm)
1	20	1	20	1	20	1	20
900	25	20	23	340	25	11,131	25
1462	30	123	30	532	27	34,699	30
1536	40	643	33	1012	30	89,037	82
2325	50	150,030	43	1673	45	90,564	95
2712	55	169,126	53	1792	50	90,911	120
2999	60	170,186	60	2230	55	90,911	200
3291	65	171,092	63	2993	60	N.A. ^*^	N.A. ^*^
3620	70	173,286	65	3050	65	N.A. ^*^	N.A. ^*^
3929	75	176,425	69	3075	70	N.A. ^*^	N.A. ^*^
4678	80	186,522	76	3080	80	N.A. ^*^	N.A. ^*^
14,798	85	193,219	85	3082	100	N.A. ^*^	N.A. ^*^
14,839	90	196,832	200	3082	200	N.A. ^*^	N.A. ^*^
14,898	100	N.A. ^*^	N.A. ^*^	N.A. ^*^	N.A. ^*^	N.A. ^*^	N.A. ^*^
14,898	200	N.A. ^*^	N.A. ^*^	N.A. ^*^	N.A. ^*^	N.A. ^*^	N.A. ^*^

^*^ The specimen has fractured into halves.

**Table 7:** Detection of fatigue crack extension using strain gauges positioned at different measurement locations. Crack length was determined from strain retraction, while the corresponding number of cycles was obtained from synchronized time data.

Strain Gauge	Vertical Position (mm)	Peak Strain (με)	Detected Time (s)	Loading Cycle	Strain Gauge	Vertical Position (mm)	Peak Strain (με)	Detected Time (s)	Loading Cycle
1	20	71	52	1	6	70	36	570	2879
2	30	98	140	125	7	80	36	615	3270
3	40	54	243	677	8	100	28	759	4075
4	50	56	305	1077	9	120	82	954	5273
5	60	59	444	1873	10	140	51	954	5273

**Table 8:** Quantitative deviations between crack size measurement methods. The relative difference was calculated with respect to the crack size measured by the compliance method, where compliance was defined as the slope of the *P*–CMOD curve (green lines in Figure 16).

Specimen	Compliance (Slope of CMOD/P)		Strain Gauges or Visual Observation		Compliance (CMOD/P)max		Compliance (ΔCMOD/ΔP)	
	Loading Cycle	Crack Size (mm)	Crack Size (mm)	Relative Difference (%)	Crack Size (mm)	Relative Difference (%)	Crack Size (mm)	Relative Difference (%)
S-0.80-0.1-1 (strain gauges)	125	21.4	30	40.1	20.5	4.1	20.5	4.4
	677	23.4	40	71.1	22.5	3.9	21.7	7.1
	1077	24.7	50	102.7	24.5	0.8	25.3	2.7
	1873	25.8	60	132.5	26.9	4.1	24.3	5.9
	2879	30.8	70	127.2	30.8	0.1	26.8	13.0
	3270	31.4	80	155.0	32.8	4.6	28.0	10.8
	4075	37.8	100	164.6	39.4	4.2	33.1	12.4
Avg.	/	/	/	113.3	/	3.1	/	8.0
S-0.80-0.5-5 (visual observation)	20	24.8	23	7.1	21.0	15.1	20.4	17.4
	123	24.8	30	21.2	21.5	13.1	20.6	16.9
	643	25.6	33	29.0	21.9	14.6	20.6	19.6
	150,030	30.4	43	41.5	43.0	41.5	24.5	19.3
	169,126	35.6	53	49.0	51.3	44.1	28.3	20.3
	170,186	36.1	60	66.2	52.0	44.0	28.8	20.1
	171,092	36.2	63	74.2	52.2	44.2	28.9	20.0
	173,286	36.1	65	79.9	53.5	48.1	29.4	18.6
	176,425	37.5	69	83.9	55.7	48.5	30.2	19.5
	186,522	45.6	76	66.8	71.1	55.9	37.2	18.5
	193,219	48.8	85	74.3	78.2	60.4	40.0	18.1
Avg.	/	/	/	53.9	/	39.0	/	18.9
S-0.85-0.5-4 (visual observation)	11,131	27.6	25	9.4	27.7	0.5	23.1	16.4
	34,699	28.1	30	6.7	34.8	23.8	23.3	17.2
	89,037	34.3	82	138.9	41.5	20.9	28.3	17.5
	90,564	43.2	95	120.1	53.9	25.0	35.8	17.1
Avg.	/	/	/	68.8	/	17.5	/	17.1

**Table 9:** Slope *n* and intercept lg*C* for each series of specimens and their combinations according to Paris Law. Results based on regression analysis and simple algebraic averages are provided.

Case	Series of Specimens	Maximum Stress Level *S*_max_	Stress Ratio *R*	Number of Specimens	Regression-Based Method			Algebraic Average Method			
					Slope *n*	Intercept lg*C*	Coefficient of Determination R^2^	Slope *n*		Intercept lg*C*	
								Mean	COV	Mean	COV
1	C1	0.80	0.1	6	13.453	−22.462	0.633	16.140	0.36	−26.560	−0.33
2	C2	0.80	0.5	2	15.080	−21.695	0.536	15.018	0.01	−21.495	−0.04
3	C3	0.85	0.1	9	8.765	−14.808	0.464	16.784	0.45	−27.001	−0.43
4	C4	0.85	0.5	7	12.654	−18.200	0.254	15.851	0.42	−21.554	−0.42
5	C5	0.90	0.5	3	18.258	−24.979	0.420	18.465	0.38	−24.563	−0.38
6	C1,C2	0.80	0.1,0.5	8	5.347	−9.741	0.354	15.860	0.31	−25.293	−0.31
7	C3,C4	0.85	0.1,0.5	16	3.846	−7.105	0.269	16.375	0.42	−24.618	−0.43
8	C1,C3	0.80,0.85	0.1	15	10.661	−17.902	0.505	16.526	0.40	−26.824	−0.38
9	C2,C4,C5	0.80,0.85,0.90	0.5	12	14.184	−20.155	0.327	16.365	0.36	−22.296	−0.36
10	C1~C5	0.80,0.85,0.90	0.1,0.5	27	3.779	−7.105	0.231	16.455	0.38	−24.812	−0.38
11	C2,C5	0.80,0.90	0.5	5	15.670	−22.043	0.405	17.086	0.31	−23.336	−0.29
12	C1,C2,C3,C5	0.80,0.85,0.90	0.1,0.5	20	4.413	−8.123	0.271	16.666	0.37	−25.952	−0.36

**Table 10:** Slope *n*′ and intercept lg*C*′ for each series of specimens and combinations of series according to Forman Equation. Results are based on simple algebraic averages.

Case	Series of Specimens	Maximum Stress Level *S*_max_	Stress Ratio *R*	Number of Specimens	Slope *n*′		Intercept lg*C*′	
					Mean	COV	Mean	COV
1	C1	0.80	0.1	6	14.183	0.42	−22.212	−0.41
2	C2	0.80	0.5	2	13.461	0.01	−18.418	−0.05
3	C3	0.85	0.1	9	14.849	0.53	−22.668	−0.53
4	C4	0.85	0.5	7	14.368	0.45	−18.577	−0.48
5	C5	0.90	0.5	3	17.768	0.31	−22.565	−0.33
6	C1,C2	0.80	0.1,0.5	8	14.002	0.36	−21.263	−0.37
7	C3,C4	0.85	0.1,0.5	16	14.639	0.48	−20.878	−0.51
8	C1,C3	0.80,0.85	0.1	15	14.583	0.48	−22.486	−0.47
9	C2,C4,C5	0.80,0.85,0.90	0.5	12	15.067	0.37	−19.547	−0.38
10	C1~C5	0.80,0.85,0.90	0.1,0.5	27	14.798	0.42	−21.180	−0.44
11	C2,C5	0.80,0.90	0.5	5	16.045	0.28	−20.906	−0.27
12	C1,C2,C3,C5	0.80,0.85,0.90	0.1,0.5	20	14.948	0.43	−22.091	−0.43

**Table 11:** Comparison of slope and intercept between this study and literature data for ordinary plain concrete with strength grades ranging from 30 to 60 MPa. Only crack propagation properties measured using SENB specimens subjected to three-point bending were compared.

Reference	*f*_cu_ (MPa)	Water/Cement Ratio	Dimension ^1^ *L* × *H* × *B* (mm)	*S*/*H*	Number of Specimens	α_0_	Stress Ratio *R*	Slope ^2^ *n* or *n*′	Intercept ^2^ lg*C* or lg*C*′	*K*_IC_ ^2^ $(MPa\cdot \sqrt{mm}$)	Analytical Model
[5]	30.4	0.52	550 × 200 × 100	2.5	>10	0.20	0.1,0.2,0.23	3.13 2.05	−8.98 −7.21	74.95	Paris Forman
[8]	27.6	0.51	1524 × 152 × 51	8.9	14	0.51	0.1,0.2,0.3	3.13	−6.49	36.68	Paris
[9]	32.8	0.60	406/203/102 × 127/76/38 × 38	2.5	6	0.17	0	10.6	−16.76	N.A. ^3^	Paris
[11]	55.6	0.41	516/223/96 × 215/93/40 × 40	2.4	18	0.30	0.06	8.35	−13.49	N.A. ^3^	Paris
[13]	44.9	0.52	800/550 × 300/200 × 100	2.5	31	0.20~0.50	0.1	−18.62α_0_ + 13.08	−1.54–1.5*n*	74.63	Paris
Current study	65.4	0.46	600 × 200 × 100	2.5	36	0.10	0.1,0.5	16.46 14.80	−24.81 −21.18	63.25	Paris Forman

^1^ More than one specimen size was reported for the size effect study. ^2^ When necessary, the values have been converted after considering the unit of N-mm, and only algebraic average values are reported in the table. ^3^ The data were not reported in the literature.

## Data Availability

The raw data supporting the conclusions of this article will be made available by the authors on request.

## References

[1] Chen H.T., Zhan X.W., Zhu X.F., Zhang W.X. (2022). Fatigue evaluation of steel-concrete composite deck in steel truss bridge—A case study. Front. Struct. Civ. Eng..

[2] Chen H.T., Li D.W., Zhu X.F., Zhang W.X. (2023). Short-term shrinkage stress in deck concrete of rail-cum-road truss bridge. Case Stud. Constr. Mat..

[3] Wang Y.P., Li J. (2021). A two-scale stochastic damage model for concrete under fatigue loading. Int. J. Fatigue.

[4] Riyar R.L., Mansi, Bhowmik S. (2023). Fatigue behaviour of plain and reinforced concrete: A systematic review. Theor. Appl. Fract. Mech..

[5] Wu Z.M., Zhao G.F., Huang C.K. (1995). Investigation of fatigue fracture properties of concrete. China Civ. Eng. J..

[6] Wu Z.M., Song Y.P., Zhao G.F., Huang C.K., Dong C. (1997). Crack propagation of concrete under fatigue loading. J. Dalian Univ. Technol..

[7] Perdikaris P.C., Calomino A.M., Chudnovsky A. (1986). Effect of fatigue on fracture-toughness of concrete. J. Eng. Mech-ASCE.

[8] Baluch M.H., Qureshy A.B., Azad A.K. Fatigue Crack Propagation in Plain Concrete. Proceedings of the SEM/RILEM International Conference on Fracture of Concrete and Rock.

[9] Bažant Z.P., Xu K.M. (1991). Size effect in fatigue fracture of concrete. ACI Mater. J..

[10] Bažant Z.P., Schell W.F. (1993). Fatigue fracture of high-strength concrete and size effect. ACI Mater. J..

[11] Kirane K., Bažant Z.P. (2016). Size effect in Paris law and fatigue lifetimes for quasibrittle materials: Modified theory, experiments and micro-modeling. Int. J. Fatigue.

[12] Zhang X. (2020). Size Effect of Fatigue Fracture of Mode I Crack in Concrete. Master’s Thesis.

[13] Lu X.J. (2000). Study on Fatigue Fracture and Size Effect of Concrete. Master’s Thesis.

[14] Dubey S., Kumar B., Ray S. (2024). Heterogeneity induced fracture characterization of concrete under fatigue loading using digital image correlation and acoustic emission techniques. Int. J. Fatigue.

[15] Sain T., Kishen J.C. (2007). Prediction of fatigue strength in plain and reinforced concrete beams. ACI Struct. J..

[16] Slowik V., Plizzari G.A., Saouma V.E. (1996). Fracture of concrete under variable amplitude fatigue loading. Mater. J..

[17] Shah S.G., Kishen J.M.C. (2012). Use of acoustic emissions in flexural fatigue crack growth studies on concrete. Eng. Frac. Mech..

[18] Brake N.A., Chatti K. (2017). Equivalent crack, fracture size effect, and cohesive stress zone of plain concrete under quasi-static and variable high-cycle fatigue loading. J. Mater. Civ. Eng..

[19] Carlesso D.M., de la Fuente A., Cavalaro S.H.P. (2019). Fatigue of cracked high performance fiber reinforced concrete subjected to bending. Constr. Build. Mater..

[20] Niu Y.F., Huang H.L., Wei J.X., Jiao C.J., Miao Q.Q. (2022). Investigation of fatigue crack propagation behavior in steel fiber-reinforced ultra-high-performance concrete (UHPC) under cyclic flexural loading. Compos. Struct..

[21] Kumar B., Sharma A., Ray S. (2024). Characterization of crack-bridging and size effect on ultra-high performance fibre reinforced concrete under fatigue loading. Int. J. Fatigue.

[22] Chen C., Chen X.D., Guo S.S. (2019). Experimental study on acoustic emission characteristic of fatigue crack growth of self-compacting concrete. Struct. Control Health Monit..

[23] Shi D.D., Chen X.D., Shen N., Liu S.S., Li S.T. (2019). Experimental and analytical study on fatigue crack propagation of pervious concrete. J. Test. Eval..

[24] Vicente M.A., Ruiz G., Gonzalez D.C., Minguez J., Tarifa M., Zhang X.X. (2018). CT-Scan study of crack patterns of fiber-reinforced concrete loaded monotonically and under low-cycle fatigue. Int. J. Fatigue.

[25] Carlesso D.M., Cavalaro S., de la Fuente A. (2021). Flexural fatigue of pre-cracked plastic fibre reinforced concrete: Experimental study and numerical modeling. Cem. Concr. Compos..

[26] Liu M.Y., Lu J., Jiang W.H., Ming P. (2023). Study on fatigue damage and fatigue crack propagation of rubber concrete. J. Build. Eng..

[27] Miarka P., Seitl S., Bílek V., Cifuentes H. (2022). Assessment of fatigue resistance of concrete: S-N curves to the Paris’ law curves. Constr. Build. Mater..

[28] Wu Z.M., Zhao G.F., Song Y.P., Huang C.K. (2000). An investigation on crack propagation process in concrete under fatigue loading by means of photoelastic coating. J. Exp. Mech..

[29] Radhika V., Kishen J.M.C. (2024). Bayesian analysis of acoustic emission data for prediction of fatigue crack growth in concrete. Theor. Appl. Fract. Mech..

[30] Li D., Huang P., Chen Z., Yao G., Guo X., Zheng X. (2020). Experimental study on fracture and fatigue crack propagation processes in concrete based on DIC technology. Eng. Fract. Mech..

[31] Jia M.D., Wu Z.M., Yu R.C., Zhang X.X. (2022). Experimental investigation of mixed mode I-II fatigue crack propagation in concrete using a digital image correlation method. Eng. Fract. Mech..

[32] Hillerborg A., Modéer M., Petersson P.E. (1976). Analysis of crack formation and crack growth in concrete by means of fracture mechanics and finite elements. Cem. Concr. Res..

[33] Hillerborg A. (1991). Application of the fictitious crack model to different types of materials. Int. J. Fract..

[34] Paris P., Erdogan F. (1963). A critical analysis of crack propagation laws. ASME J. Basic Eng..

[35] Forman R.G., Kearney V.E., Engle R.M. (1967). Numerical analysis of crack propagation in cyclic-loaded structures. ASME J. Basic Eng..

[36] Chen H.T., Sun Z.Y., Zhang X.W., Fan J.H. (2023). Tensile fatigue properties of ordinary plain concrete and reinforced concrete under flexural loading. Materials.

[37] Chen H.T., Zhuo Y.F., Li D.W., Huang Y. (2024). Fracture toughness of ordinary plain concrete under three-point bending based on double-k and boundary effect fracture models. Materials.

[38] Islam R.U., Khalil J., Hanif A. (2025). Fatigue performance of ultra-high-performance concrete (UHPC): A critical review. J. Build. Eng..

[39] Siregar A.P.N., Rafiq M.I., Mulheron M. (2017). Experimental investigation of the effects of aggregate size distribution on the fracture behaviour of high strength concrete. Constr. Build. Mater..

[40] Beygi M.H.A., Kazemi M.T., Nikbin I.M., Vaseghi A.J., Rabbanifar S., Rahmani E. (2014). The influence of coarse aggregate size and volume on the fracture behavior and brittleness of self-compacting concrete. Cem. Concr. Res..

[41] Guan J.F., Song Z.K., Zhang M., Yao X.H., Li L.L., Hu S.N. (2021). Concrete fracture considering aggregate grading. Theor. Appl. Fract. Mech..

[42] Meng Q.C., Hu L., Wan D., Li M.J., Wu H.J., Qi X., Tian Y.D. (2023). Image-based concrete cracks identification under complex background with lightweight convolutional neural network. KSCE J. Civ. Eng..

[43] Kim B., Natarajan Y., Preethaa K.R.S., Song S., An J., Mohan S. (2024). Real-time assessment of surface cracks in concrete structures using integrated deep neural networks with autonomous unmanned aerial vehicle. Eng. Appl. Artif. Intell..

[44] Carpinteri A., Spagnoli A. (2004). A fractal analysis of size effect on fatigue crack growth. Int. J. Fatigue.

[45] Carpinteri A., Paggi M. (2007). Self-similarity and crack growth instability in the correlation between the Paris’ constants. Eng. Fract. Mech..

[46] Ray S., Kishen J.C. (2011). Fatigue crack propagation model and size effect in concrete using dimensional analysis. Mech. Mater..

[47] Simon K.M., Kishen J.M.C. (2017). A multiscale approach for modeling fatigue crack growth in concrete. Int. J. Fatigue.

[48] Bhowmik S., Ray S. (2018). An improved crack propagation model for plain concrete under fatigue loading. Eng. Fract. Mech..

